# Barriers and enablers for health system reform

**DOI:** 10.3389/frhs.2026.1887541

**Published:** 2026-09-07

**Authors:** Danitza Chavez-Montoya, Caitlin Main, Kavyashree Satish, Borna Müller, Jennifer Gill, Panos Kanavos

**Affiliations:** 1 Medical Technology Research Group, Department of Health Policy, London School of Economics and Political Science, London, United Kingdom; 2 External Affairs International, F. Hoffmann-La Roche Ltd, Basel, Switzerland

**Keywords:** health system reform, healthcare workforce, NCD, screening, service delivery decentralisation

## Abstract

**Introduction:**

Health systems worldwide face escalating pressure from the growing burden of non-communicable diseases and population ageing, as well as chronic workforce shortages, infrastructure limitations, and structural misalignment between care models designed for acute illness and the long-term needs of complex patients. Many countries are pursuing comprehensive transformation efforts including shifting care closer to home, enhancing early disease detection and addressing workforce challenges. This study aims to identify examples of transformation across these domains, including specifically service delivery decentralisation, workforce transformation and enhancement of screening and diagnostic services for early detection and treatment - and determine common barriers and enablers in order to make recommendations for improvement.

**Methods:**

A mixed-methods approach, combining a scoping literature review and semi-structured one-on-one interviews was utilised. Searches of peer-reviewed literature were performed in EMBASE, Global Health, and Ovid MEDLINE databases. The Rayyan platform was used for systematic management of references, deduplication, and screening. Titles and abstracts were independently reviewed by two members of the research team before full-text review and data extraction. 153 peer-reviewed papers and 35 grey literature reports were analysed, whilst 17 expert stakeholders, including policy experts, academics, health policy experts and clinicians from six countries were interviewed.

**Results:**

67 transformation case studies were included. Of these, 36 were focused on service delivery decentralisation, 16 on screening and 12 were related to workforce adaptation. A final set of 34 barriers and 27 enablers was identified and grouped empirically into six areas including: political, governance and policy; funding, incentives & resource allocation; workforce capacity, capability & culture; system integration, structure and care coordination; digital infrastructure, technology and data; communication, engagement, and change management.

**Discussion:**

Finally, a set of 25 recommendations, across seven groups, are made for policy makers and other relevant stakeholders. These findings provide practical, evidence-based insights to guide policymakers in designing scalable, context-sensitive health system transformation strategies.

## 1 Introduction

Health systems and governments globally are under increasing pressure from a combination of complex and intersecting challenges that threaten their sustainability and effectiveness. The most significant of these is the increasing burden of Non-communicable Diseases (NCDs), primarily cardiovascular diseases (CVD), cancers, diabetes (DM), and chronic respiratory diseases, which collectively constitute for approximately 74% of all deaths globally (exceeding 43 million deaths in 2021) (1). This burden is compounded by ageing populations, with the proportion of people over 60 projected to nearly double from 12% to 22% by 2050 (2), placing increasing strain on all healthcare services. Additionally the rising prevalence of mental health issues, often co-morbid with physical issues, adds additional burden on healthcare system resources as well as a layer of complexity, demanding specialized, integrated care (3).

These trends are particularly pronounced in OECD countries, where NCDs account for the vast majority of deaths and a substantial share of overall disease burden (4). Much of this burden stems from CVD (28%) and cancer (21%), followed by chronic respiratory diseases (6%) and diabetes (3%) (5) Furthermore, improvements in survival rates for single conditions have contributed to rising levels of multimorbidity^1^, where individuals live longer with multiple chronic conditions. This growing complexity of care needs challenges health systems that remain largely configured around single-disease models (6–8).

Health system's capacity to address population and patient needs in increasingly constrained by competing budgets, chronic workforce shortages, and structural inefficiencies (9). A primary driver of this systemic strain is that contemporary health models remain structurally optimized for acute, episodic care, making them fundamentally ill-equipped to handle the complex requirements of an aging demographic increasingly challenged by multimorbidity (10). Addressing these systemic failures demands a strategic pivot beyond incremental budgeting toward a comprehensive transformation of the health system. This transformation requires a fundamental shift in how resources are allocated; moving from passive funding to what Kutzin et al. define as strategic purchasing, where financial flows are deliberately linked to performance and outcomes (11). Such reforms are increasingly recognised as essential for improving efficiency and aligning incentives with population health needs (4).

Achieving this transformation requires sustained attention to key pressure points within the health system. At least three specific pillars are relevant.

First, decentralization of service delivery. Insufficient investment in primary and community-based services has led many health systems to rely heavily on hospital care (5). Addressing this imbalance requires a deliberate shift in care delivery, from high-cost hospital settings toward community- and home-based models, which can alleviate pressure on acute sectors while improving patient adherence and outcomes (4). Achieving this transition requires a strategic reallocation of resources towards primary care, alongside the adoption of digital health solutions, including telemedicine and virtual wards (4, 12). Strengthening primary and community services may also help position primary care as the first point of contact within the health system (13).

Second, workforce transformation and upskilling. Persistent workforce shortages have become a major constraint on health system reform, limiting the ability of systems to deliver coordinated, preventive, and community-based care. In Europe, the shortage of doctors, nurses, and midwives was estimated at 1.2 million in 2022, while over one-third of doctors and one-quarter of nurses were already aged over 55, underscoring the scale and urgency of the capacity challenge (14). Addressing these challenges requires a shift away from traditional workforce models organised around professional silos and toward multidisciplinary, team-based approaches that can better support integrated and person-centred care (15). Achieving this transition will require sustained investment in training, retention, and working conditions, alongside supportive policy, funding, and incentive structures that enable collaboration across roles and settings (14, 15). Strengthening the workforce in this way may help health systems improve coordination, expand access, and deliver care more effectively in the face of rising chronic disease burden. Evidence suggests that aligning workforce incentives with team-based care delivery is critical to sustaining such reforms (11).

Third, strengthening -screening and early diagnosis. Health systems that rely predominantly on late-stage diagnosis remain locked into reactive and often more costly models of care. Delays in diagnosis are associated with lower survival, greater treatment burden, and higher costs, whereas early detection enables more effective and timely intervention (16). Addressing this imbalance requires a deliberate shift in resources and service design toward earlier detection, stronger diagnostic pathways, and more timely referral and treatment initiation. A multi-faceted approach is required, integrating national screening plans and health literacy campaigns to ensure broad population engagement (17). Risk stratification approaches can further support the targeting of high-risk populations and improve efficiency in resource allocation (18). In addition, investment in diagnostic infrastructure and clinical decision support tools can improve diagnostic accuracy and timeliness (12). When combined with streamlined referral procedures, these interventions can identify complications before they escalate into high-cost medical crises, ultimately preserving system capacity and improving long-term population health.

Together, these three strategic pillars provide a structured lens through which to analyse health system transformation. This research examines health system reform across OECD countries through comparative case study analysis. The study aims to identify common policy barriers and enablers across three domains: (1) health service delivery decentralisation, defined as the reorientation of care delivery from hospital-based to primary and community settings; (2) workforce transformation, encompassing policy responses to workforce supply constraints and evolving skill requirements; and (3) early screening, diagnosis, and treatment, involving systematic efforts to detect and manage disease at earlier stages. Drawing on this analysis, the study seeks to develop a set of prioritised policy recommendations to support health system sustainability and performance.

## 2 Methodology

We followed a three-pronged approach combining a brief targeted review, a scoping review to identify case studies and semi-structured one-on-one interviews. The semi-structured interviews were designed to complement and triangulate findings from the literature review, rather than to achieve theoretical saturation.

### 2.1 Targeted literature review

Prior to the scoping literature review, a brief, targeted review was performed of both peer-reviewed and grey literature in order to understand the key drivers, challenges, and priorities shaping health system transformation. Searches were conducted using a combination of academic databases and general search engines to capture relevant policy reports (e.g., WHO, OECD, and national strategies) and recent review articles. The search was iterative and purposive, focused on identifying seminal frameworks, commonly cited reports, and recurring themes rather than achieving comprehensive coverage. Findings from this review allowed the development of the keywords used in the scoping review search strategy, semi-structured interview guide and a number of analytical endpoints.

### 2.2 Scoping review

A comprehensive scoping review was conducted to identify evidence across three key thematic areas: service delivery decentralisation, healthcare workforce and early screening/diagnosis/treatment. Searches of peer-reviewed literature were performed in EMBASE, Global Health, and Ovid MEDLINE databases. Eligible records were limited to English-language publications available in full text and published between January 2015 and December 2024. Studies of all empirical types were considered, including original research articles, systematic and narrative reviews, case studies, and clinical evaluations. Inclusion criteria included full-text studies published in English that reported at least one barrier or enabler of healthcare transformation in one of the three areas of interest, from a country of interest, with priority given to OECD member countries and selected additional focus countries. Studies were required to focus on one of the NCD disease areas of interest, including oncology/haematology, neurology, ophthalmology, immunology/respiratory disease, or cardiovascular/metabolic conditions, and to report information relevant to innovation promotion, such as healthcare delivery efficiency, adoption of new technologies, financial or funding incentives, digital health and technology investment, clinical trial activity, or HTA and regulatory processes.

Exclusion criteria encompassed clinical trials lacking a health-service delivery or transformation component; studies focused solely on biological, pharmacological, or laboratory outcomes; and non-empirical sources such as opinion or perspective pieces, editorials, and conference abstracts and proceedings. The detailed search strategy and full search terms are provided in the Supplementary Materials.

Search results were imported into the Rayyan platform (19) for systematic management of references, deduplication, and screening. A two-stage screening process was applied. First, titles and abstracts were independently reviewed by two members of the research team to determine relevance to the predefined thematic areas and eligibility for full-text assessment. Discrepancies were discussed and resolved through consensus, using the inclusion and exclusion criteria as reference. Second, full-text review and data extraction were conducted using a structured Excel template. Specifically, information was collected on publication details (including title, author, year, journal, publication type, and source), study characteristics (objective, abstract, case study status, country and income group focus), and clinical or thematic scope (disease area and specific areas of focus such as oncology, cardiovascular disease, or chronic conditions). In addition, data were extracted on any key sub-dimensions of transformation for each of the three main focus areas. For example, for decentralisation sub-categories included alternative care models, community care investment and patient centric care. For screening, sub-categories included national screening plans, improved infrastructure for early diagnosis and treatment, improved referral procedures. Finally, for workforce, sub-categories included investment in upskilling, retention of workforce, use of multidisciplinary teams and workforce planning. The extraction also captured reported barriers, outcomes or impact measures, study findings, and recommendations, alongside identified enablers. Backward and forward citation tracking (snowballing) was performed to identify additional relevant publications that were not identified through the searches.

Complementary searches of grey literature were undertaken to capture evidence from policy documents, programme evaluations, institutional reports and other relevant sources. These searches included the OECD Library, WHO Global Health Observatory, and the Commonwealth Fund databases, among others. Similar inclusion and exclusion criteria and data extraction template were applied to grey literature sources to ensure consistency and comparability across evidence types.

The literature review provided a foundation for understanding existing interventions and documented outcomes, while highlighting the gaps and contextual variations across health systems.

### 2.3 Stakeholder interviews

The preliminary literature review informed the development of a semi-structured interview guide, which was tailored to reflect the specific expertise and professional background of each stakeholder. This adaptive approach ensured that the interviews remained relevant and allowed for deeper exploration of context-specific insights. Questions centred on the three focus areas: service delivery decentralisation, workforce adaptation and screening and early treatment – with triggers, barriers and enablers of redesign being of primary interest. Additionally, stakeholders were asked about the cross-cutting role of digital health, future policy recommendations and experience of health reform case studies in their countries of expertise. The semi-structured interview guide is outlined in the Supplementary Material.

Interviewees were selected from the researchers' networks based on their expertise and strategic relevance to the research. As mentioned, the objective of the stakeholder interviews was to validate, contextualise, and refine existing insights, rather than to exhaustively capture all possible perspectives. As such selecting informed experts with relevant experience was important. Potential interviewees were invited by email and received informed consent forms before the interview. Interviews took place either virtually or face-to-face, depending on logistics and participant preference. All interviews were audio-recorded and transcribed verbatim with participants’ consent.

Interviews captured general perspectives on health system transformation as well as examples of case studies, typically reflecting the interviewee's country of expertise. These examples were treated as embedded case studies for analysis. In these situations, relevant supporting literature on the specific case studies was identified and used in the analysis of case studies (Section 2.3.2). Data from interview transcripts were systematically extracted into a structured Excel grid capturing key characteristics of any case studies discussed, including intervention type, policy context, implementation approach, and reported barriers and enablers.

Given the focused sample size, analysis followed a structured thematic approach rather than a formal coding framework. All team members independently reviewed the extracted data and identified key barriers and enablers suggested by interviewees. These were then compared across cases to identify recurring patterns and common themes. Differences in interpretation were discussed within the research team until agreement was reached, allowing themes to be refined and consolidated.

This process received ethics approval from the LSE Research Ethics Review Board. To support validation of findings, a subset of stakeholders were invited to a roundtable discussion on the 22nd May, 2025 on the periphery of the 78th World Health Assembly Meeting in Geneva, Switzerland. This session was used to review preliminary findings, validate emerging themes, and refine interpretation through stakeholder feedback. Together with the literature review this ensured that case study identification is both evidence-based and grounded in lived practice.

### 2.4 Case study selection

Case studies were identified through both the literature review and stakeholder interviews. Case studies reported in papers identified via the literature review were included if they met the inclusion criteria (case study from a country of interest, with priority given to OECD member countries and selected additional focus countries, focusing on one of the NCD disease areas of interest - oncology/haematology, neurology, ophthalmology, immunology/respiratory disease, or cardiovascular/metabolic conditions – with the transformation process focusing on one of the three key areas of interest – service delivery decentralisation, screening and early treatment or workforce adaptation). Additional case studies identified during the interview process were followed up with targeted literature searches to verify and document the examples, and to inform the analysis of reported impacts, barriers and enablers. Case studies were then assessed using the structured framework described below.

### 2.5 Framework for analysis

To analyse the identified health system redesign case studies, a structured framework was developed and applied. This framework outlined the pathway from initial transformation/redesign *triggers* through to the ultimate *policy objectives*, highlighting the dynamic interplay between the strategic interventions, influencing factors, and system-level outcomes. This structure provides a coherent lens through which case studies can be examined and compared.

The redesign process is initiated by system transformation triggers or drivers—contextual opportunities that generate momentum for change. Such triggers may arise from epidemiological shifts, policy reforms, technological innovations, fiscal pressures, or infrastructure deficiencies. Whilst these triggers establish awareness and direction, they do not guarantee successful transformation; effectiveness depends on the presence of enabling conditions and the active mitigation of barriers, which operate at both system and intervention levels. Frequently, these triggers are themselves responses to pre-existing systemic obstacles that hinder necessary transformation. These challenges stem from the inherent characteristics of the health system or from the manner in which health care is planned and delivered. For example, siloed budget lines can prevent the pooling of funds across departments, resulting in fragmented health financing. It is also important to note that the influence between triggers and obstacles is bidirectional: triggers may highlight system barriers prior to intervention selection, and systemic obstacles may themselves prompt the need for change before the appropriate intervention is chosen. Such constraints restrict policymakers’ ability to reallocate resources swiftly in times of crisis, irrespective of whether the intervention involves a new digital health platform or a rural staffing initiative. These barriers operate at a broader system level and are not confined to individual strategic interventions.

**Figure 1:**
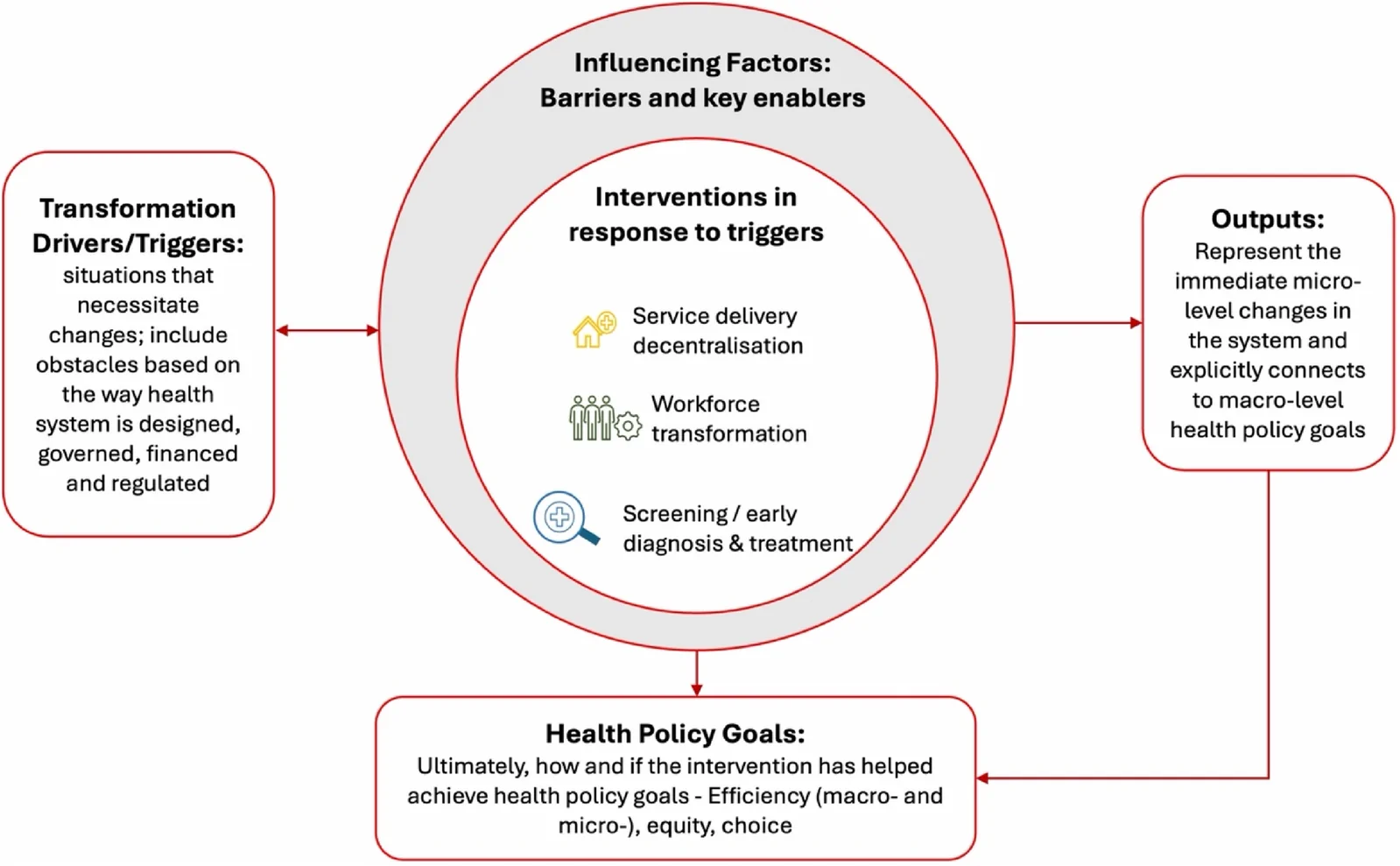
Conceptual framework for health system transformation, arranged around a central circle. On the left, transformation drivers and triggers, situations that necessitate change, including obstacles arising from how the health system is designed, governed, financed and regulated. A double-headed arrow links these to the centre, indicating influence in both directions. The centre comprises two concentric circles: an inner circle of interventions made in response to triggers, illustrated by service delivery decentralisation, workforce transformation, and screening or early diagnosis and treatment; and an enclosing outer ring of influencing factors, the barriers and key enablers through which every intervention must pass. A second double-headed arrow links the centre to outputs on the right: the immediate micro-level changes in the system, which connect explicitly to macro-level health policy goals. These lead in turn to health policy goals at the foot of the diagram, whether and how the intervention has helped achieve efficiency at both macro and micro level, equity, and choice.

Once pre-exiting obstacles are identified and considered, health systems can respond to triggers by designing and implementing strategic health interventions, as shown in Figure 1, which are central to the framework and represent the core intervention domains within the health system:

1. **Service delivery decentralisation** refers to the shifting of care closer to patients through primary care expansion, community-based models such satellite health centres and digital health platforms that could include alternative care models such as telemedicine, patient centric care, primary care and community care investment and increased provider collaboration.
2. **Workforce transformation** refers to the distribution, capacity and integration of health professionals to support coordinated care delivery, which includes investment in upskilling, enabling workforce to use technology, increasing the workforce capacity, distributing providers to underserved areas and enabling use of multidisciplinary teams
3. **Screening and early diagnosis** focuses on identifying at-risk populations to enable timely intervention and prevent disease progression. This could include initiatives such as developing national screening plans and health literacy campaigns, risk stratification, infrastructure upgrades, improved referral procedures and implementing clinical decision support tools.

While service delivery decentralisation, workforce transformation, and screening and early diagnosis are presented as distinct pillars, they function as an interdependent ecosystem. The decentralisation of care into community settings is only clinically viable if the workforce is simultaneously upskilled and redistributed to manage complex cases outside of traditional hospital environments. These two shifts, in turn, create the necessary local infrastructure to scale early diagnosis and screening initiatives. Integrated electronic health records and data-sharing platforms act as the connecting link that enables successful implementation of the change.

Each intervention operates within a context shaped by influencing factors, which are categorized here as barriers and enablers. Barriers may hinder/obstruct or distort the intended impact of an intervention, resulting in delays, partial implementation or unintended consequences. Conversely, enablers facilitate the successful activation of interventions. These factors are specific to each health intervention, and their identification may inform effective implementation strategies.

Implementing the interventions generate outputs that are immediate, measurable changes at the micro-level, such as improved screening rates, expanded service coverage, or enhanced workforce capacity. These outputs serve as intermediate indicators of progress and provide a critical link between operational activities and broader system goals. The framework subsequently connects these outputs to health system performance goals giving a clear indication of the real-world value of the system redesign and innovation. Across international frameworks there is general consensus on the broad objectives of the health system, including: health improvement, system responsiveness, equity, fair financing and efficiency, although there are multiple frameworks for addressing health system goals including the 3 “Es” Framework – efficiency, equity and equality - (20), the Health Policy Triangle - Equity, Efficiency, Cost - (21) and the triple aim - Population Health, Experience of Care, Per Capita Cost - (22). As any kind of analysis on cost of transformation processes was out of scope here, it was decided that the focus would be on the impact of transformation processes on efficiency (both micro- and macro-level), equity (including access) and choice, although it is important to note that there are other health system objectives that may be relevant in determining the effectiveness of transformation processes. These four policy objectives were further developed iteratively by synthesising themes and outcome measures identified in the literature and Supporting Materials. The four policy objectives are defined as follows, each with four sub-categories of interest:

(1) **Micro-efficiency** – The ability to improve operational efficiency at the service or organisational level focusing on processes, workflows and staff productivity. This includes care coordination personalisation, quality of care and patient experience.
(2) **Macro-efficiency** – The ability of a health system/intervention to deliver value at a system-wide level, optimising resource allocation across the entire healthcare ecosystem. Includes population coverage, health outcomes, system resilience and inter-sectoral benefits.
(3) **Equity** – The extent to which a service or intervention ensures fair access and outcomes for all population groups, particularly underserved or vulnerable communities. Geographic and socioeconomic equity along with cultural responsiveness and inclusion of vulnerable populations are included in this objective.
(4) **Choice** – The degree to which an intervention enhances patient autonomy and options, allowing individuals to make informed decisions about their care. Care preferences, provider choice, treatment decisions and service options are included here.

By explicitly connecting micro-level interventions to macro-level goals, this framework provides a coherent methodology for evaluating the effectiveness of health system transformation strategies in managing NCDs/chronic diseases. It enables a systematic analysis of how contextual triggers, strategic health interventions and influencing factors interact to produce meaningful change, and how these changes align with broader policy aspirations. Whilst the focus of the current analysis is the existence of both enablers and barriers in the various interventions considered, additional analysis examining the extent to which the identified case studies provided evidence on the impact of transformation across these four policy objectives is presented in the Supplementary Material, as it offers supportive context but is not essential to the interpretation of the primary findings. This additional analysis is intended to characterise the breadth and distribution of the evidence base, rather than to inform the core conclusions of the study and is therefore provided separately to maintain the focus and clarity of the main manuscript.

## 3 Results

### 3.1 Overview of included evidence

**Figure 2:**
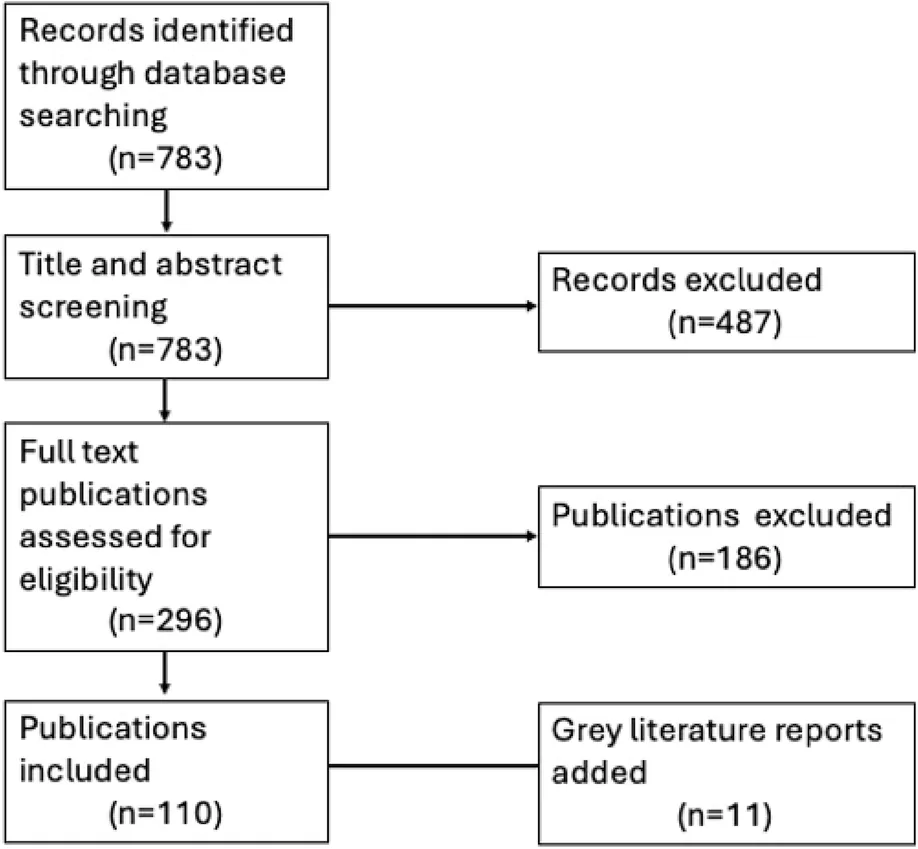
Flowchart illustrating a study selection process: from 783 records identified, 487 are excluded after title and abstract screening, 296 full texts assessed, 186 excluded, resulting in 110 included publications, with 11 grey literature reports added.

**Figure 3:**
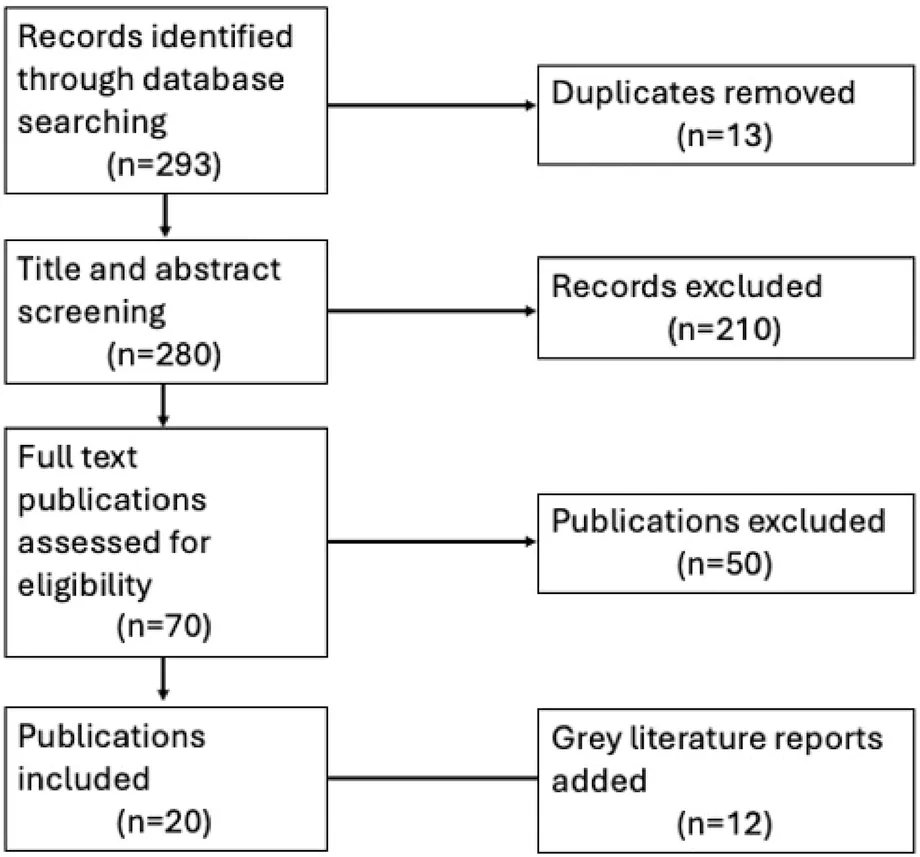
Flowchart showing records identified through database searching, with 293 total, 13 duplicates removed, 280 screened, 210 excluded, 70 texts assessed, 50 excluded, 20 publications included, and 12 grey literature reports added.

**Figure 4:**
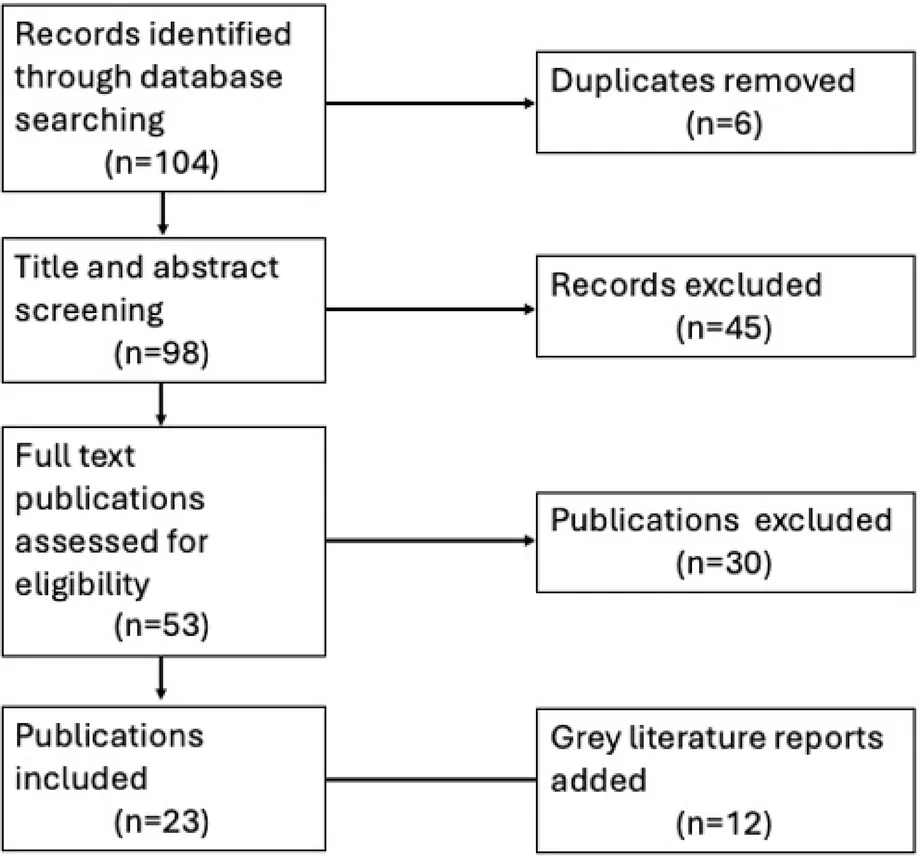
Flowchart illustration detailing a systematic review process: 104 records identified, 6 duplicates removed, 98 screened, 45 records excluded, 53 full texts assessed, 30 publications excluded, 23 publications included, and 12 grey literature reports added.

A total of 153 peer-reviewed papers and 35 grey literature reports met the inclusion criteria. Given the topic's breadth, service delivery decentralisation accounted for the largest evidence base (110 peer-reviewed articles, 11 reports), followed by health workforce transformation (23 papers, 12 reports) and screening and early diagnosis programs (20 papers, 12 reports). Figures 2–4 outline the PRISMA flow for each of the three literature searches.

Seventeen expert stakeholders were interviewed across six countries: the United Kingdom, North America, Portugal, India, Germany, and Switzerland. Stakeholders included health economists, health policy experts, clinicians, patient advocacy representatives, academics and experts in health services and healthcare leadership. Table 1 in the Supplementary Material outlines the geographic background and stakeholder affiliation or group of the stakeholders involved.

Information was extracted from the literature for 67 case studies. Of these, 36 were focused on service delivery decentralisation, 16 on screening and 12 were related to workforce adaptation (Table 1). Three case studies incorporated aspects of more than one of these focus areas. The majority of the case studies were conducted in Europe (*n* = 45), seven were focused on North America, six in Australasia. China, Singapore and Israel were represented by one case study each. The remaining six case studies focused on multiple countries. This geographic concentration has implications for generalisability, explored further in the discussion. The conditions that the case studies tended to focus on included oncology (*n* = 16), cardiovascular disease (including diabetes and chronic kidney disease) (*n* = 7), respiratory health (*n* = 4) and ophthalmology (*n* = 3). 15 case studies had multiple conditions in focus while 19 had no condition focus. The remaining three were related to mental health, reproduction and influenza.

**Table 1:** Summary of case studies identified.

Name	Country / Region	Year	Type*	Condition focus	Background	Key Findings Reported	Barriers Reported	Enablers Reported	Reference
24/7 remote rural health monitoring	Australia	2023 (Published)	D	Multiple	Provides patients with a monitoring kit for at home vital sign measurement. Includes a digital tablet for upload of health data via a secure website			Yes	(79)
Attend Anywhere / Near Me video consulting	Scotland	2016 (Implemented)	D	No condition focus	This is a video call system and service designed to support remote health and care consultation.	Yes	Yes	Yes	(38)
Benefits and Challenges of Remote Patient Monitoring as Perceived by Health Care Practitioners: A Systematic Review	Multiple Global	2023 (Published)	D	Multiple	Remote patient monitoring (RPM), or telemonitoring, offers ways for health care practitioners to gather real-time information on the physiological conditions of patients.	Yes	Yes		(41)
Canada's primary care crisis: Federal government response	Canada	Not specified	D	No condition focus	Explores options for the federal government to take a more hands-on role responding to the present crisis that are constitutionally compliant.		Yes	Yes	(80)
CLCH pilot: COPD and asthma management in patients’ homes	UK	2022 (Published)	D	COPD and asthma	Pilot to improve condition management for people living with chronic obstructive pulmonary disease (COPD) and asthma.			Yes	(81)
Clinical impact of integrated e-health system for diabetes self-management support and shared decision making (POWER2DM)	Netherlands / Spain	2023 (Published)	D	Diabetes mellitus	Developed and field-tested an e-health system (POWER2DM) that integrates medical, psychological and behavioural aspects and connected wearables to support patients and healthcare professionals in shared decision making and diabetes self-management.	Yes	Yes		(32)
Chronically ill people need well-developed primary care - a simulation model	Switzerland	Not specified	D	Multiple	Examine effect the closure of general practices have on the use of medical services, costs and patients’ health and which patient groups and regions are particularly affected.	Yes			(82)
Community Eye Centres (CEC) - Decentralisation of an Ophthalmology Service	UK	Not specified	D	Ophthalmology	The service objectives were to enable care closer to home with seamless, safe patient pathways, provide the best quality patient experience, increase cost effectiveness, and demonstrate innovation.	Yes	Yes		(28)
Decentralisation of radiation therapy - RUTE satellite radiotherapy unit	Spain	2008 (Implemented)	D	Multiple	A satellite radiotherapy unit in Spain (RUTE-Radiotherapy Unit, Terres de l'Ebre), managed by the Radiation Oncology Department at Hospital Universitari Sant Joan de Reus	Yes	Yes	Yes	(43)
Digital Transformation in Ophthalmic Clinical Care During the COVID-19 Pandemic	Multiple Global		D	Ophthalmology	Review of the digital adoption that has taken place in ophthalmic clinical care.	Yes	Yes	Yes	(30)
Effectiveness of telemonitoring to manage COPD	Multiple Global	2017	D	COPD	Review and evaluation of the effectiveness of telemonitoring to manage COPD.	Yes	Yes	Yes	(83)
Enhanced Community Care	Ireland	Not specified	D	Multiple	The HSE's Enhanced Community Care (ECC) programme is a €240 million Sláintecare initiative that expands community health services to ease hospital pressure by placing specialist teams across all health regions, each serving about 150,000 people.	Yes		Yes	(68)
Feasibility of health-related quality of life (HRQoL) assessment for cancer patients using electronic patient-reported outcome (ePRO) in daily clinical practice (QOLIBRY study)	France	2021 (Published)	D	Oncology	The QOLIBRY study looked at electronic patient-reported outcomes in those treated with systemic therapies for breast, lung or colorectal cancer at any stage. Patients were invited to complete the EORTC QLQ-C30 questionnaire and cancer-site-specific modules before each visit on tablets and/or computers in the hospital or at home.	Yes	Yes	Yes	(84)
GP Pathfinder Clinics	UK		D	No condition focus	Online GP service offering GP video consultations. Includes a number of practices in North London for face-to-face appointments			Yes	(85)
Home monitoring for women with pregnancy complications	Denmark	2021 (Implemented)	D	Reproduction	Pregnant women with complications used new telehealth equipment to avoid multiple weekly check-ups at the hospital.	Yes	Yes		(35)
Hospital at Home	UK	2021	D	Multiple	The Wandsworth and Merton Hospital at Home team, part of Central London Community Healthcare NHS Trust, provides intensive hospital-level, holistic support for serious conditions, in a patient's home.	Yes			(39)
Hospital at Home service - Spital Zollikerberg Zuhause	Switzerland	Not specified	D	Multiple	“Visit - Spital Zollikerberg Zuhause®” offers equivalent inpatient treatment at home. Patients are managed by an interprofessional team of doctors and specially trained nurses using modern telemedicine including 24/7 monitoring through modern telemedicine.			Yes	(69)
House of Health of Bettola- decentralization of cancer care	Italy	2016 (Implemented)	D	Oncology	Patients with cancer living in the Val Nure can receive care at the local community center that has an oncological suite, in addition to other community health services.	Yes			(86)
How to improve care coordination for people with chronic conditions in Switzerland?	Switzerland	Not specified	D	Multiple	The aim of the study was to define framework conditions for improved care coordination in Switzerland and to assess the potential value of new healthcare models.	Yes		Yes	(87)
Optometry First Toolkit	UK	2022 (Published)	D	Ophthalmology	Optometry First is a service commissioning and design principle establishing a co-ordinated and comprehensive primary eye care service as part of the wider eye care delivery system, reducing pressure on the hospital eye service (HES) and benefiting patients and the wider NHS.	Yes			(27)
Person-Centered Care Planning (PCCP)	USA	2022 (Published)	D	Multiple	Analysis of innovative approaches and challenges to implementing person-centered care planning (PCCP) for people living with or at risk for multiple chronic conditions	Yes	Yes	Yes	(42)
Ping An Good Doctor one minute clinics	China	2019 (Implemented)	D	No condition focus	One-minute Clinics utilise Ping An Good Doctor's mobile healthcare and AI technology, and integrate a variety of smart medical examination devices.			Yes	(88)
Remote monitoring in heart failure care	Germany	2013	D	CVD	The Telemedical Interventional Management in Heart Failure II (TIM-HF2) involved remote monitoring of weight, heart rate, heart rhythm, blood pressure, oxygen saturation and a self-rated health status transmitted daily to the person's treating clinicians.	Yes		Yes	(40)
Safe@home	Australia	2023 (Published)	D	No condition focus	The safe@home project provides patients with chronic conditions, especially those living in low-income communities, with access to daily/regular integrated primary care services through virtual care and telemonitoring.				(89)
Self-management and remote monitoring in prostate cancer	England	2015	D	Prostate Cancer	Compared outcomes of remote monitoring and self-management at a prostate cancer clinic. Participants used an online system and periodic blood samples were taken at the patient's GP practice / hospital to detect prostate-specific antigen (PSA). Results were transferred to the online system and reviewed during virtual clinics.	Yes		Yes	(36)
South Australian Child and Adolescent Virtual Urgent Care	Australia	2021	D	No condition focus	Child and Adolescent Virtual Urgent Care Service connects parents with a virtual team of highly skilled emergency doctors and nurses who can assess and provide medical advice for children, aged between 6 months and up to 18 years.	Yes		Yes	(90)
Subcutaneous administration of bortezomib for multiple myeloma at home	France	2013 (Implemented)	D	Multiple myeloma	At home treatment for all doses of Myeloma patients within each cycle - following first administration of treatment for each cycle in the outpatient unit of the Department of Hematology.	Yes		Yes	(34)
Targeted lung health check programme (TLHC)	UK	2019 (Implemented)	D	Lung cancer	The targeted lung health check programme (TLHC) offers lung health checks to participants aged 55 to 74 who are current or former smokers. Aims to improve earlier diagnosis of lung cancer, at a stage when it is much more treatable.	Yes	Yes		(72)
Telemonitoring for COPD patients	Denmark	2023 (Published)	D	COPD	Randomized controlled trial of a six-month telemonitoring service. The intervention group received home TM (Tunstall HealthCare's telemonitoring equipment) for 6 months in addition to standard COPD treatment.	Yes		Yes	(66)
The PDM-ProValue study program [4]	Germany	2018 (Published)	D	Diabetes mellitus	The integrated personalized diabetes management (iPDM) approach combines structured SMBG, use of diabetes data management software, collaborative patient-physician communication, and support of therapeutic decision-making in an iterative, 6-step, structured intervention process.	Yes			(33)
The right care in the right place (JZOJP)	Netherlands	2018 (Published)	D	Multiple	Relocating healthcare, for instance, from the hospital to the general practitioner (GP) or from the GP to other healthcare providers or to citizens themselves. Relocation of care is expected to reduce costs and manpower shortages				(91)
Treating people with acute mental illnesses at home	Switzerland	2016 (Implemented)	D	Mental health	The Ticino Cantonal Psychiatric Clinic replaced an acute inpatient ward with a new service for psychiatric treatment and care for patients at home in the Bellinzona and Valli district.	Yes		Yes	(92)
Trial to monitor chemotherapy side effects remotely	Multiple Global	2016	D	Oncology	The European multicentre randomised controlled trial (eSMART) evaluated the impact of remote monitoring of chemotherapy side effects on patients’ symptom burden, anxiety, work limitations, supportive care needs and quality of life.	Yes			(93)
Virtual outpatient clinic development: using the NASSS framework	UK	2019 (Published)	D	No condition focus	Barts Health NHS Trust and the University of Oxford are working together to develop video-based remote consulting as part of business as usual.	Yes	Yes		(65)
Virtual wards	UK	2023 (Published)	D	Multiple	Assessment of Virtual Wards in the UK		Yes		(37)
We4You- Integrated medical practice	Switzerland	2024 (Published)	D	No condition focus	“We4You” offers a classic medical practice with a ‘walk-in principle’. Doctors are supported by specialized nursing professionals who also assess and treat diseases independently (in consultation).			Yes	(94)
Digitalisation in Estonia towards disease prevention and early detection	Estonia	2019 (Implemented)	D / S	Multiple	An online platform using AI-based automatization to improve efficiency and effectiveness of NCD screening programme		Yes		(29)
Virtual clinics for people with respiratory symptoms	England	2018	D / S	Long-term respiratory conditions	A referral management offers advice, virtual clinics, preclinic investigations and follow-up in the community for people living with chronic diseases such as asthma, chronic obstructive pulmonary disease and sleep apnoea. GPs refer patients to specialists for appropriate investigations. Respiratory specialists are then responsible for reviewing patient data and triaging patients without meeting them in person.	Yes			(95)
Blood pressure trial launches in Bunnings NSW	Australia	2023 (Published)	S	CVD	Aims to identify more people with high blood pressure and raise community awareness by placing SiSU Health stations in 30 Bunnings stores across New South Wales, Australia. Adults who uncover high blood pressure are referred for medical care or lifestyle guidance and can track their progress with a free app.			Yes	(59)
C the Signs	UK	2021	S	Oncology	A new diagnostic tool that helps to detect cancer in its earliest and most curable stages. Designed to be used within a GP consultation, C the Signs supports clinicians in recommending what investigations or referrals the patient may need.	Yes		Yes	(57)
Cost-Effectiveness Analysis of Colorectal Cancer Screening Strategies in Singapore: A Dynamic Decision Analytic Approach	Singapore	2004 (Published)	S	Colorectal cancer	Five commonly used colorectal cancer screening strategies are being evaluated – Guaiac Fecal Occult Blood (FOBT), Immunochemical Fecal Occult Blood (FOBT(IMM)), Double Contrast Barium Enema (DCBE), Flexible Sigmoidoscopy (FSIG) and Colonoscopy (COL) to determine the most cost effective	Yes			(96)
Deployment of a multi-tissue AI-based quality control system in routine clinical workflow (GALEN)	Israel	2022 (Published)	S	Oncology	AI-based system in multi-tissue pathology diagnostic systems	Yes			(97)
Diabetes Prevention Programme (NHS DPP)	UK	2016 (Implemented)	S	Diabetes mellitus	Healthier You NHS Diabetes Prevention Programme identifies people at risk of developing type 2 diabetes and refers them onto a nine-month, evidence-based lifestyle change programme. The Healthier You programme is available both as a face-to-face group service and as a digital service.	Yes		Yes	(52)
Digital Health check	UK	2023 (Implemented)	S	Multiple	The initiative will deliver 1 m checks in the first four years. Tens of thousands of cases of hypertension are expected to be identified and hundreds of strokes and heart attacks prevented. Patients will be able to access the check via a mobile phone, tablet or computer.			Yes	(98)
Early diagnosis for Breast Cancer	Ukraine	2019 (Implemented)	S	Breast cancer	Early breast cancer diagnosis programme, based on the rapid identification of cancer in patients who have symptoms of the disease and require rapid full diagnosis follow up.	Yes			(12)
Global Flu View	Multiple Global	2018	S	Influenza	Three examples of participatory surveillance systems – Flu Near You in North America, Influenzanet in Europe, and Flu Tracking in Australia and New Zealand – have recently collaborated to develop Global Flu View, a shared platform for aggregation and dissemination of crowdsourced data on influenza-like illness	Yes			(99)
HPV screening in Scotland	Scotland		S	Oncology	Analysis of the effectiveness of the bivalent human papillomavirus (HPV) vaccine in preventing invasive cervical cancer	Yes			(100)
Lessons learnt from the implementation of a colorectal cancer screening programme for lynch syndrome in a tertiary public hospital	Spain	2016 (Implemented)	S	Colorectal cancer	Analysed the established screening programme for Lynch Syndrome (the first cause of inherited colorectal cancer (CRC)	Yes			(67)
Lung cancer screening programme in Croatia	Croatia	2020 (Implemented)	S	Lung cancer	Croatia introduced a national lung cancer screening programme in October 2020, becoming the first country in the European Union (EU) to do so. The screening programme is targeted at people aged 50 to 75, with a smoking history of 30 pack-years, who currently smoke or have stopped smoking within the past 15 years.	Yes			(53)
Moldova: Radiology and Diagnostic Imaging Center	Moldova	2011 (Implemented)	S	Multiple	The Moldovan Ministry of Health used public-private partnerships (PPPs) to modernize diagnostic imaging and radiology services for public patients at the Republican Hospital in Chisinau.	Yes	Yes	Yes	(101)
NHS blood pressure checks at the barbers to prevent killer conditions	UK	2023 (Published)	S	CVD	The NHS is expanding blood pressure checks available in local communities including barbershops, mosques and at a dominoes club, as part of a major drive to prevent strokes and heart attacks.	Yes		Yes	(58)
Opt-in and opt-out self-sampling HPV screening strategies	Netherlands	2017 (Implemented)	S	Cervical cancer	Self-collection and an ‘opt-out’ approach for HPV screening and cervical cancer prevention.	Yes	Yes		(60)
Screening Centre Integration: HPV self-sampling at mammography clinics	Denmark	2014 (Implemented)	S	Cervical cancer	Leveraging the high participation rate in breast cancer (BC) screening as an opportunity to offer self-sampling kits to nonparticipants in cervical (CC) and colorectal (CRC) cancer screening.	Yes			(55)
USPSTF Colorectal Cancer Screening Recommendation and Uptake for Individuals Aged 45 to 49 Years	USA	2021 (Implemented)	S	Colorectal cancer	The US Preventive Services Task Force (USPSTF) issued a recommendation for CRC screening in adults aged 45 to 49 years, mandating insurance coverage for this preventive service.	Yes	Yes		(102)
Development of a comprehensive prevention strategy for CKD in France	France	2002	S / W	CKD	Comprehensive strategy for preventing and managing CKD, slowing the progression of the illness to end-stage kidney disease, and, when necessary, providing access to blood dialysis technology or arranging and carrying out kidney transplants. Key components include prevention and patient education programmes, early diagnosis and treatment measures, online patient monitoring, thorough data collection and sharing, and quality/satisfaction evaluation questionnaires.	Yes			(103)
Evaluation of the Effectiveness of Health Care Provider Incentive Programs in Oregon	USA	2018 (Implemented)	W	No condition focus	This study evaluates Oregon's health care provider incentives and informs efforts to achieve health equity and increase access to culturally responsive care in urban and rural underserved areas of the state.	Yes	Yes		(104)
Interventions for health workforce retention	Multiple Global	2021 (Published)	W	No condition focus	This review looked at the benefit and impact of strategies focused on nurse retention.				(50)
Americas Health Corps – Fuerza de Salud de las Americas	Americas	2022 (Implemented)	W	No condition focus	Americas Health Corps aimed to help the region prevent, prepare for and respond to future pandemic threats and other public health emergencies, while ensuing the equitable delivery of health care services to remote, vulnerable and marginalized communities. It aimed to train 500,000 public health workers throughout the region over the next five years.			Yes	(47)
BeWell project - skills development initiative	Multiple European	2022	W	No condition focus	The BeWell project aims to map digital and green skill needs in the health workforce and create a Europe-wide skills strategy through a new Blueprint Alliance. Using co-creation and extensive stakeholder engagement, it will involve target groups in defining challenges and shaping solutions for developing and integrating project results.				(105)
Health workforce New Zealand	New Zealand	2009 (Implemented)	W	No condition focus	Health Workforce New Zealand (HWNZ) is charged with providing national leadership for the development of the country's health and disability workforce and with the overall responsibility for planning and development of the health workforce to ensure that it is fit for purpose. HWNZ uses of a wider range of planning methods to develop broader workforce intelligence variables. It led the planning and development of New Zealand's health and disability workforce to ensure it was “fit for purpose” and could meet future health needs.				(46)
HOPE - Hospitals of EurOPE	Europe	1981 (Implemented)	W	No condition focus	The exchange programme offers those with managerial responsibilities across the healthcare system a unique opportunity to exchange time with another EU member state for four weeks, followed by an international meeting for all participants.			Yes	(106)
HR Action Plan	Canada	2022	W	No condition focus	In November 2022, the Manitoba government launched a $200 million Health Human Resource Action Plan to invest in distinct programs and approaches to better support nurses, doctors, allied health professionals and support staff across the province. The focus was on three core pillars: RETAIN, TRAIN, and RECRUIT.				(51)
IFMSA exchange program	Canada	Not specified	W	No condition focus	The International Federation of Medical Student Associations (IFMSA) represents, connects and engages a network of 1.3 million medical students in 130 countries around the globe. Each year, more than 15,000 medical students have the opportunity to explore health care delivery and health systems in different cultural and social settings through IFMSA exchanges.				(107)
Nurse-led care models reduce unplanned hospital admissions	Switzerland	2016	W	No condition focus	A nurse-led care model for chronically ill residents of Swiss nursing homes with the intention of reducing unnecessary hospitalisations. The model was designed in such a way that it can be used in real life in nursing homes of different sizes and with different levels of equipment.	Yes		Yes	(108)
Pharmacy First	UK	2024	W	Multiple	Pharmacy First service enables patients to be referred into community pharmacy for a minor illness or an urgent repeat medicine supply. It includes the supply of appropriate medicines for 7 common conditions including earache, sore throat, and urinary tract infections, aiming to address health issues before they get worse.			Yes	(109)
Skill mix in Poland	Poland	2021 (Published)	W	No condition focus	Analyses the impact of assigning new competences to selected groups of medical professionals – e.g. nurses, physiotherapists - to optimise the mix of skills in healthcare.		Yes		(110)
Workforce Incentive Program – Rural Advanced Skills Stream	Australia	2024 (Implemented)	W	No condition focus	Payment of doctors for work in a variety of remote and rural areas. The Rural Advanced Skills Stream rewards investment in specialist qualifications and advanced skills and encourages more doctors to work in a variety of settings using these skills in regional, rural and remote areas.				(49)

* D refers to case studies focusing on service delivery decentralisation, S to those focusing on screening, W to those focusing on workforce adaptation and D/S to those that have elements of both service delivery decentralisation and screening.

Findings outlined below integrate evidence from the literature and interviews, with interview data used to contextualise, validate and extend documented barriers and enablers.

### 3.2 Reform area specific insights

#### 3.2.1 Service delivery decentralisation

The service delivery decentralisation evidence base is rich, with 110 included peer-reviewed articles and 11 reports. Included sources explore community hospitals, nurse-led interprofessional models, alongside case studies exploring virtual wards and out of hospital frailty pathways.

Several decentralisation strategies were explored, including alternative care models (i.e., telemedicine or virtual care wards), patient-centric care, investment in primary and community care, and increased collaboration between providers. For example, community hospitals in Italy's Emilia-Romagna region were used to deliver integrated care closer to where people live, with organisational responsibility assigned to nurses and strong links to primary and community care (23). In England, case studies highlighted community responses and frailty services that combine paramedic triage, advanced clinical practitioners, care home teams and social care partners to keep older people at home wherever possible, instead of defaulting to the emergency department (24, 25). In contrast to case studies centred around workforce improvements or enhanced screening and early treatment, those focusing on service delivery decentralisation were more likely to report barriers and enablers as outlined below.

Analysis across the international cases shows a consistent set of system-level enablers that support the decentralisation of care from hospitals into community, primary care, and home-based settings. A central enabler is the use of structured referral and access mechanisms that act as formal gateways for redistributing activity away from hospital front doors. Single Points of Access (SPoA), referral advisory services, and central triage functions enable specialist expertise to be applied upstream while allowing care delivery to occur in lower-acuity settings. In the virtual respiratory clinics, a cloud-based referral advisory service enabled remote specialist triage and redirection into community pathways, contributing to reduced outpatient demand while maintaining patient safety (26). Similar referral governance structures underpin Optometry First, where a formal ophthalmic SPoA ensures that patients are directed to community optometrists or hospital services based on clinical risk rather than default hospital referral (27). In ophthalmology decentralisation initiatives such as Community Eye Centres, central referral oversight enabled redistribution of routine activity while preserving specialist capacity for complex cases (28).

A second, widely observed enabler is digital infrastructure that enables clinical work to be performed without physical co-location. Across many cases, decentralisation depends not merely on teleconsultation but on integrated digital ecosystems that support referral, diagnostics, monitoring, and escalation. Estonia's national digital health system illustrates how a mature, interoperable infrastructure can embed prevention and early detection directly within primary care, enabling risk stratification and personalised prevention outside hospital settings (29). In ophthalmology, electronic referrals, image sharing, and virtual review enable assessment and follow-up for glaucoma, cataract, and urgent eye conditions in community practices rather than hospital clinics (27, 30). Similarly, digital platforms underpin remote monitoring and virtual supervision in Hospital at Home services, COPD telemonitoring programmes, heart failure monitoring, and virtual wards, allowing hospital-level oversight while care is delivered at home (31–34). Overall, this enabler was identified in over 54% of the decentralisation case studies reviewed, underscoring its central role in enabling decentralised models of care.

Decentralisation is further enabled by workforce redesign and task redistribution, particularly the expansion of non-physician roles and multidisciplinary team-based care. Many cases demonstrate that shifting care out of hospitals requires not only technology but explicit reallocation of clinical responsibilities. In We4You, specialised nursing staff independently assess and treat patients within an integrated primary care model, supported by mobile diagnostics and specialist input when needed (31). In digital chronic disease programmes such as POWER2DM and PDM-ProValue, decentralisation occurs through supported self-management, device-enabled data capture, and clinician oversight conducted remotely, allowing ongoing disease management to shift away from hospital clinics (32, 33). Nurse-led and multidisciplinary models are also central to home-based cancer care, where visiting nurses trained in subcutaneous chemotherapy administration deliver treatment safely at home, with clear escalation pathways back to hospital (34). These models highlight how decentralisation depends not only on technology but also on workforce redesign and the redistribution of clinical responsibility. They also highlight the interconnectedness of the areas of focus looked at here. This enabler was also observed across a large share of case studies, highlighting its widespread role in enabling decentralised care models.

Governance structures and formalised cross-sector collaboration further enable decentralisation. Denmark's nationally coordinated adoption of home fetal monitoring illustrates how system-wide alignment reduces duplication and ensures that community and home-based services can safely substitute for hospital-based care (35). Likewise, the shared governance model in Optometry First establishes consistent standards, accreditation, and feedback mechanisms across multiple community providers, supporting quality assurance as care is decentralised (27). These governance arrangements enable decentralisation by reducing variation and reinforcing accountability across settings.

Clear clinical protocols, risk stratification frameworks, and escalation pathways represent another critical enabler. Across several cases, decentralisation was possible because eligibility criteria and thresholds for escalation were explicitly defined (34). In optometry-led ophthalmology pathways, structured risk classification determines which patients can be managed entirely in the community and which require specialist review (27). In prostate cancer survivorship programmes, remote monitoring and self-management are coupled with rapid re-entry to specialist clinics when predefined thresholds are crossed (36). Virtual wards and remote monitoring programmes similarly rely on protocolized deterioration pathways and clearly assigned clinical responsibility to ensure safety outside hospital walls (37)

Patient acceptance and engagement further function as enabling conditions, which legitimises decentralised models and supports sustainability. Home-based fetal monitoring, chronic disease self-management programmes, and hospital-at-home services are sustained in part because patients perceive greater convenience, continuity, and autonomy compared with hospital-based care (32, 35, 38). This acceptance reduces resistance to care relocation and supports the redistribution of services into community and home settings.

Despite these enablers, the cases also reveal persistent barriers that constrain the scale and durability of decentralised care. Workforce pressures are among the most frequently reported barriers. Many initiatives depend on highly trained nurses, specialist oversight, or extended clinical availability, which can exacerbate existing workforce shortages. Hospital-at-home services and remote monitoring programmes frequently report increased workload, need for rapid skill expansion, and difficulty sustaining staffing levels alongside traditional services (39, 40). In the Nemo Healthcare model, staff shortages and limited training capacity slowed implementation despite clear clinical benefits (35).

Digital inequities and interoperability limitations also constrain decentralisation. While digital access enables distributed care, uneven connectivity, limited digital literacy, and fragmented information systems undermine reliability and equity. Several cases report difficulties integrating remote monitoring data into electronic health records, requiring manual workarounds and increasing administrative burden (38, 41). Lack of interoperability between health and social care systems limits the effectiveness of person-centred care planning and weakens community-based follow-up (42). Similarly, digital exclusion among older adults, disadvantaged populations, and rural communities further restricts the reach of decentralized. As a result, although digital infrastructure is a primary enabler of decentralisation, it also constitutes one of its most significant barriers, as highlighted across over half of the decentralisation case studies.

Resource dilution and infrastructure constraints represent another barrier. In decentralised ophthalmology services, high-cost diagnostics remain centralised because they are too expensive to replicate across multiple community sites, requiring some patients to travel back to hospital and limiting full pathway decentralization (28). Similar constraints appear in radiotherapy hub-and-spoke models, where dependence on central planning infrastructure restricts the extent of decentralisation despite local treatment delivery (43).

Finally, implementation complexity and coordination burden frequently limit scalability. Decentralised care models introduce new logistical demands, including coordination across providers, data governance, supply chains, and training requirements. Home chemotherapy required explicit coordination costs, drug transport logistics, and administrative support that are not present in traditional outpatient pathways (34).

Taken together, the cases demonstrate that decentralisation of care is enabled by structured access mechanisms, digital infrastructure, workforce redesign, safety backstops, aligned governance, and patient acceptance, while being constrained by workforce capacity limits, digital fragmentation, resource concentration, and implementation burden.

#### 3.2.2 Workforce adaptation

The workforce evidence base comprised of 23 peer-reviewed studies and 12 grey literature reports. Across the sources, shortages of health professionals, imbalances in skill-mixes and an uneven distribution between urban and rural areas were consistently highlighted as key constraints on health system performance.

Five broad categories of workforce interventions were identified, including, (i) investment in upskilling and reskilling healthcare professionals, (ii) retention and wellbeing measures, (iii) deployment of digital technologies to support staff, (iv) expansion of workforce capacity and redistribution to underserved areas, and (v) the development of multidisciplinary teams and task-sharing team models. European Commission reports described large-scale upskilling initiatives, such as Sweden's recovery and resilience plan for investment to train and upskill long-term care staff and EU-wide programs like the Pact for Skills, which focus on digital competencies for health workers (44). The same report documents retention strategies including significant wage increases for doctors, dentists and pharmacists, and targeted financial incentives to attract and retain staff in underserved areas, showcased by pay rises and rural incentives in Latvia.

Several sources explored multidisciplinary team models as core responses to multimorbidity and complex social needs. In France, successive governments have implemented various initiatives to combat ‘medical deserts’, which are severely underserved areas where residents face major barriers to access needed health services. The predominant response has been to create multidisciplinary health homes, which allow GPs and other primary care workers to work in the same location, supported by financial incentives. As a result of these efforts, 2,500 homes were set up by the end of 2023, although this was still considered insufficient to resolve the issue (14). The same OECD report has also summarised the EU funded TaSHI project, which has developed curricula, a guidebook and recommendations to support task-shifting across five EU countries, showcasing how structured delegation of tasks and expanded roles for nurses and other professionals, such as pharmacists, are being used to increase productivity and resilience. In parallel, many countries throughout the OECD have expanded the scope of nurses, often in advanced or nurse practitioners roles, alongside creating new roles such as physician assistants to improve access, continuity and quality of care while reducing pressure on GPs (14, 45).

Across the cases reviewed, workforce-related health system transformation was enabled by a combination of policy instruments aimed at strengthening capacity, redistributing skills, and improving coordination across professional groups. At the same time, persistent structural, governance, and implementation barriers constrained the extent to which workforce reforms translated into sustained system transformation.

A central enabling factor was the presence of explicit national or regional workforce policy frameworks with dedicated leadership and planning mandates. Centralised workforce governance structures enabled a shift away from fragmented, employer-led decision-making towards more coordinated and forward-looking approaches. In New Zealand, the establishment of Health Workforce New Zealand (HWNZ) as a national entity responsible for workforce planning and development created a mechanism for system-wide coordination and longer-term workforce strategy (46). Similarly, large-scale regional initiatives such as the Americas Health Corps created a shared policy framework across countries, explicitly linking workforce development to health system resilience and equity objectives (47).

Investment in workforce upskilling and expanded scopes of practice also emerged as a key enabler of transformation. Several initiatives highlighted the role of structured training and regulatory reform in enabling new or expanded professional roles. In Switzerland, nurse-led models in nursing homes were supported by targeted training that enabled nurses to assume expanded responsibilities, alongside leadership development and coaching mechanisms (48). In New Zealand, regulatory reforms under the Health Practitioners Competence Assurance Act facilitated overlapping scopes of practice and the introduction of nurse practitioner roles, supporting task redistribution and more flexible team-based care (46). In Australia, the Workforce Incentive Program – Rural Advanced Skills embedded upskilling within a financial incentive framework by rewarding doctors for acquiring and using advanced procedural and non-procedural skills in rural and remote settings (49). This pattern was observed across many of the workforce case studies reviewed, indicating its consistent role in enabling workforce adaptation.

Retention-oriented workforce policies were particularly impactful when they combined education with financial, organisational, and psychosocial supports. Distributed education models supporting rural health workers to undertake further university study, through paid tuition, flexible schedules allowing concurrent employment, local delivery, and tailored academic support, were associated with high retention and low turnover across multiple studies involving 392 nurses (50). In one rural U.S. medical centre, annual turnover of licensed practical nurses declined from 16.8% to 6.8% following implementation of such a programme (50). Comparable approaches were observed in Manitoba, where financial incentives, mental health supports, licensing reimbursement, and workplace safety measures were implemented as part of a comprehensive health workforce action plan (51).

Financial incentives targeting workforce distribution, particularly towards rural, remote, or underserved areas, were another consistent enabler. Policy mechanisms included location-based payments, scaled incentives by remoteness, and targeted recruitment schemes. The Australian WIP – Rural Advanced Skills programme explicitly tied payments to Modified Monash (MM)3–7 locations and scaled incentives to encourage service delivery in more remote areas (49). The Americas Health Corps similarly emphasised training and equipping public health workers for deployment in remote, vulnerable, and marginalised communities, embedding equity objectives within workforce policy design (47). In Manitoba, remote location incentives and the creation of a provincial float pool were described as mechanisms to improve coverage in areas experiencing uneven staffing pressures (51).

Digital tools and technology also played an enabling role by supporting workforce productivity, reach, and coordination. At a regional level, the Americas Health Corps leveraged the PAHO Virtual Campus to deliver large-scale training across geographically dispersed settings, enabling workforce development without reliance on physical infrastructure (47). In Manitoba, initiatives such as the Virtual Emergency Care and Transfer Resource Service (VECTRS) and expanded virtual psychotherapy enabled clinicians to access specialist advice and extend service delivery while mitigating workforce pressures (51).

The promotion of multidisciplinary and team-based models of care also functioned as an important enabler of transformation. Several initiatives explicitly sought to move away from profession-specific silos towards integrated workforce models. In New Zealand, workforce planning approaches such as the Workforce Service Forecast emphasised clinician-led, patient-centred scenarios built around teams rather than individual professions (46). In Swiss nursing homes, the nurse-led care model enhanced interprofessional cooperation and coordination between nurses and physicians, contributing to improved care processes (48). Manitoba's action plan similarly included measures to introduce medical assistants, coordinators, and support roles to reduce administrative burdens and enable clinicians to focus on complex care (51).

Improvements in workforce intelligence, data, and horizon scanning were identified as enabling conditions for system-level transformation. In New Zealand, HWNZ implemented comprehensive workforce forecasting models incorporating demographics, retirement patterns, and migration trends, alongside qualitative intelligence such as scope-of-practice and plasticity analyses (46). These tools supported a shift from static supply projections towards adaptive planning under uncertainty, enabling alignment between future service models and workforce capabilities.

Despite these enabling conditions, substantial barriers to workforce-related health system transformation were consistently reported. A pervasive constraint was the lack of sustained, coherent workforce governance, particularly in contexts characterised by decentralisation or repeated institutional restructuring. In New Zealand, extended periods of workforce policy neglect during health system reforms resulted in the loss of planning structures and increased fragmentation as workforce decisions were devolved to individual employers, leading to duplication and short-termism (46).

Administrative and implementation burdens also limited workforce transformation. Programs often required complex eligibility criteria, documentation, or multiple application processes, potentially reducing uptake. In Australia, separate applications for different incentive streams and requirements to meet minimum service thresholds were identified as structural constraints embedded within program design (49). More broadly, innovation pilots frequently struggled to diffuse beyond initial sites, remaining localised despite positive evaluations, as observed in New Zealand (46). This barrier was identified in over 60% of the workforce case studies reviewed, indicating that administrative and implementation complexity is a recurrent constraint on workforce transformation.

Fragmentation and entrenched professional silos constituted another major barrier. Discipline-based training, regulation, and organisational cultures often limited the adoption of multidisciplinary and flexible workforce models. In New Zealand, conflicting priorities among workforce actors and resistance to new planning approaches, particularly scenario-based and uncertainty-embracing methods, were described as constraining integrated planning and service redesign (46).

Persistent mal-distribution of the workforce remained a barrier even in the presence of targeted incentives. Rural and underserved areas continued to experience shortages of highly skilled professionals, complicating the implementation of uniform care models. In New Zealand, uneven access to general practitioners and higher-skilled nursing personnel in rural areas was highlighted as an ongoing constraint on integrated care delivery (46). Similar challenges were implicit in the design of rural incentive programs in Australia and recruitment-focused initiatives in the Americas, reflecting the structural difficulty of retaining staff in remote settings despite financial supports (47, 49).

Administrative and implementation burdens also limited workforce transformation. Programs often required complex eligibility criteria, documentation, or multiple application processes, potentially reducing uptake. In Australia, separate applications for different incentive streams and requirements to meet minimum service thresholds were identified as structural constraints embedded within program design (49). More broadly, innovation pilots frequently struggled to diffuse beyond initial sites, remaining localised despite positive evaluations, as observed in New Zealand (46). This barrier was identified in over 60% of the workforce case studies reviewed, indicating that administrative and implementation complexity is a recurrent constraint on workforce transformation.

Finally, workforce exhaustion and morale issues emerged as barriers to sustained transformation. High workloads, burnout, and dissatisfaction undermined retention and limited capacity for change. In Manitoba, the need for explicit mental health and burnout supports was acknowledged as a response to workforce strain, highlighting the fragility of reform efforts in overstretched systems (51). Similarly, pandemic-related pressures exposed longstanding weaknesses in workforce resilience, even where enabling policies existed (47).

#### 3.2.3 Screening and early treatment

Screening and early diagnosis evidence was drawn from 20 peer-reviewed sources and 12 grey literature reports covering a mix of cancer and non-communicable disease (predominantly diabetes) programs. Across the included studies, six main categories of screening interventions tended to be identified: national or regional screening strategies, health literacy campaigns, risk-stratified or targeted screening approaches, improved infrastructure to enable early diagnosis and treatment, implementation of clinical decision support tools for early diagnosis, and lastly, efforts to improve referral procedures. National or sub-national programs were often combined with community outreach or primary care engagement activities.

Screening case studies observed tended to be more likely to report impact than the case studies in the service delivery decentralisation and workforce-related examples. For instance, the NHS Diabetes Prevention Programme resulted in a 7% reduction in the number of new diagnoses of Type 2 diabetes in England between 2018 and 2019, and those completing the nine-month scheme reduced their chance of getting Type 2 diabetes by more than a third (52). Similarly, the lung cancer screening programme in Croatia has been shown to result in an observed stage shift to earlier detection in lung cancer. By 2023 the programme had achieved screening coverage of 80%, screening more than 26,000 participants and identifying over 300 cancers (53). This is likely because screening-based interventions tend to produce quantifiable, near-term results whilst those associated with decentralisation and workforce related transformation examples may need long-term embedment and multi-year behaviour change to observe results. It may also be ‘easier’ to attribute results to screening programs which tend to be linear and controlled vs. workforce and decentralisation reforms which may include many confounding factors and may not be required to report outcome metrics.

Across screening programmes and disease areas, a consistent set of system-level enablers support the expansion of early detection while shifting activity upstream and outside traditional hospital settings. A dominant enabling factor is the use of trusted, pre-existing points of contact within the health system to anchor screening delivery. Programmes that integrate screening into routine interactions with primary care or established prevention pathways demonstrate higher reach and operational feasibility. In the lung cancer screening programme in Croatia, recruitment is led directly by primary care physicians, enabled by universal patient registration and high annual contact rates with general practice, estimated at around 90 percent of the population. This model allows systematic identification and referral of eligible individuals without reliance on centralised invitation systems, embedding screening into routine care rather than creating parallel structures (53, 54). Similarly, the NHS Diabetes Prevention Programme relies on primary care identification and referral of individuals at high risk, with almost one million people referred since launch, illustrating how existing care relationships can be leveraged at scale for preventive screening and intervention (52).

A related enabler is the strategic integration of screening within established national programmes that already benefit from high participation and institutional trust. In Denmark, HPV self-sampling has been embedded within the national cervical cancer screening programme rather than introduced as a stand-alone initiative, ensuring alignment with existing call-recall systems, quality assurance mechanisms, and follow-up pathways. Targeting self-sampling initially to non-responders enabled measurable coverage gains without disrupting clinician-based screening, contributing to a sustained reduction in the pool of long-term non-attenders (55). A comparable approach is observed in cluster-randomized trials that leveraged attendance at breast cancer screening, where participation exceeds 80 percent, to offer cervical and colorectal cancer screening to women overdue for those programmes, demonstrating how high-trust platforms can be used to expand coverage efficiently (56).

Digital and administrative infrastructure that supports eligibility assessment, referral, and follow-up emerges as a second critical enabler. Screening initiatives that are underpinned by interoperable registries, electronic medical records, and scheduling platforms are better able to identify eligible populations, track participation, and ensure continuity along the diagnostic pathway. In Croatia's lung cancer programme, access to electronic health records and a national digital scheduling platform enables real-time identification of eligible individuals and streamlined referral to one of 16 certified screening centres (53). In Denmark, integrated population registries and automated call–recall systems allow precise targeting of non-attenders and monitoring of follow-up compliance across multiple screening programmes (55). Similarly, the C the Signs programme demonstrates how embedding AI-enabled decision support directly into GP electronic medical records can strengthen early cancer detection without adding workflow burden, achieving a reported 12.3% increase in cancer detection rates without additional resource use (57).

Decentralisation of screening delivery into community and non-clinical settings constitutes another major enabling mechanism, again confirming the close interlinkage between the three focus areas studied here. Moving screening closer to everyday environments reduces logistical and psychosocial barriers associated with clinical appointments and expands reach to populations less engaged with routine healthcare. Blood pressure screening initiatives in England and Australia illustrate how community pharmacies, barbershops, mosques, and retail environments can function as effective screening sites, supported by clear referral pathways into formal care. In England, the expansion of blood pressure checks into community venues was enabled by dedicated funding and integration within integrated care systems, facilitating coordination across NHS services, local authorities, and voluntary organisations (58). In New South Wales, self-operated digital kiosks placed in hardware stores enabled rapid, private screening without dependence on clinical staff, illustrating how technology can support scalable, low-resource screening delivery (59).

Self-sampling modalities further enable decentralisation by reducing reliance on clinician-delivered screening. Across cervical cancer screening programmes, HPV self-collection addresses structural and personal barriers that limit participation, particularly among under-screened women. Evidence from multiple European settings shows that opt-out self-sampling strategies are especially effective in increasing uptake among women who do not routinely attend screening, while PCR-based HPV testing on self-collected samples demonstrates comparable analytical performance to clinician-collected samples when validated assays are used (60). When integrated within national programmes, and supported by structured follow-up pathways, self-sampling can also alleviate pressure on primary care capacity by reducing the number of routine consultations required for screening (55).

Financial and reimbursement alignment plays a critical enabling role in sustaining screening delivery at scale. Programmes that explicitly recognise and compensate additional workload are more likely to secure clinician engagement and avoid displacement of routine care. In Croatia, primary care physicians are reimbursed for the work involved in identifying and referring eligible individuals for lung cancer screening, directly addressing workload concerns and supporting sustained participation (53). In Moldova, tariff reform and payment guarantees enabled a public–private partnership to mobilise private capital for diagnostic imaging, expanding access to early diagnosis for approximately 100,000 people annually without requiring upfront public investment (61). These examples highlight the importance of aligning financial incentives with screening objectives to ensure operational viability.

Despite these enabling conditions, several recurring barriers constrain the effectiveness and equity of screening-based transformation. Limitations in follow-up capacity and system readiness also constrain screening impact. While decentralised and self-sampling approaches increase initial participation, their effectiveness depends on timely diagnostic confirmation and treatment. In colorectal cancer screening strategies evaluated in Singapore, invasive follow-up procedures such as colonoscopy introduce risks, costs, and capacity constraints that can undermine the net benefit of screening, particularly when population compliance is variable. Similar concerns arise in HPV self-sampling pathways, where additional triage steps following positive results increase the risk of loss to follow-up if systems are not adequately prepared (60). This barrier was identified in approximately 50% of the case studies reviewed, underscoring its recurrent role in constraining system transformation.

Another prominent barrier is unequal reach to populations disengaged from existing services. Screening models anchored in primary care contact or attendance at other screening programmes inherently exclude individuals with limited healthcare engagement, often those facing socioeconomic, cultural, or geographic barriers. Even in Croatia, where GP attendance is high, reliance on primary care recruitment may miss individuals with infrequent contact, while interventions leveraging breast cancer screening attendance do not address deeper social determinants of non-participation (53, 56).

Workforce dependence and capacity constraints constitute a further barrier. Programmes that rely heavily on clinician time and engagement are vulnerable to workload pressures, staffing shortages, and variable uptake. In Croatia, lung cancer screening places substantial responsibility on individual primary care physicians for eligibility assessment and appointment booking, creating potential bottlenecks under conditions of workforce strain (53). Similarly, digitally enabled tools such as C the Signs face variability in onboarding and utilisation across practices, limiting consistent system-level impact despite strong evidence of effectiveness (57).

Finally, digital inequities and infrastructure gaps remain a cross-cutting barrier. Screening models that rely on digital tools, apps, or electronic records may inadvertently exclude individuals with limited digital access or literacy. Uneven IT maturity across providers also constrains scalability and transferability, particularly in settings without interoperable registries or automated referral systems. Without targeted mitigation strategies, these constraints risk reinforcing existing inequities in screening access and outcomes.

## 4 Discussion

This study addresses a notable paucity in the literature by analysing the enablers and barriers of large-scale health system adaptations, providing an empirical basis for bolstering resilience against escalating global demands. Furthermore, we introduce a simple framework to evaluate the impact of these efforts across micro- and macro-efficiency, equity, and patient autonomy and choice. Addressing the mounting burden of NCDs and chronic conditions necessitates an urgent, system-level transformation that moves beyond incremental adjustments toward comprehensive resilience. The current analysis provides a critical lens through which to understand the mechanics of this transition. A significant number of barriers and enablers were identified via a literature review of transformation examples (Table 2). Information from the stakeholder interview process confirmed the importance of many of these barriers and enablers and allowed us to develop empirical groupings of the barriers and enablers offering a practical basis for identifying actionable levers for policy and implementation.

**Table 2:** Common barriers and key enablers.

Area of focus	Barriers	Enablers
Political, Governance and Policy	• Short-term political interests override strategic health needs • Infrastructure and policy favour hospitals over community or preventive care • Lack of shared governance and leadership results in poor coordination across sectors • Issues around data ownership, privacy, and policy inconsistency, particularly regarding electronic health records (EHR) • Reliance on historical spending patterns (funding vertical, disease-specific programs) limits resources for integrated health initiatives. • Limited involvement of other governmental stakeholders (e.g., Ministries of Finance, Education) in cross-cutting initiatives	• Political commitment and leadership with a focus on the wider political ecosystem and aspirations for a healthy and prosperous society • Long-term, bipartisan health strategies following a clear national vision and strategy that survive political cycles • Cohesive and high-quality policy informed by evidence and data
Funding, Incentives & Resource Allocation	• Short-term funding models with little long-term planning or sustainable investment • Historical underfunding, especially for prevention, primary care, and workforce development • Reimbursement gaps for preventive care and digital tools • Lack of coordinated funding between federal, state, and insurance bodies • Billing and classification issues causing confusion between capital and operational spending (e.g., digital investments)	• Sustained and safeguarded investment in health sufficient to address both current and future demands. • Proactive financial planning aligning allocations to short- and long-term health objectives. • Dedicated investment in infrastructure, digital health, and workforce innovation, plus targeted transformation funds. • Performance-linked payments to encourage quality improvement and continuity of care. • Shift from volume-based to value-based and risk-adjusted reimbursement mechanisms. • Provider incentives aligned with health outcomes, prevention, and coordinated care delivery.
Workforce Capacity, Capability & Culture	• Underutilisation of professionals and limited task sharing • Insufficient funding for training, leadership, and capability building • Limited workforce planning, resulting in mismatches between system needs and training outputs • Resistance to change among healthcare staff	• Mid-level managers and local leaders with clear mandates and operational tools to drive transformation • Multidisciplinary care teams fully utilising nurses, pharmacists, paramedics, and community health workers • Patient navigators to help patients access effective treatment and care
System Integration, Structure and Care Coordination	• Poor integration between primary, secondary, social care, and diagnostics, leading to fragmented care coordination • Weak or absent integration of social care, causing poor continuity of care • Lack of holistic patient pathways, with societal determinants and behaviours insufficiently addressed • Misaligned financial incentives distorting care delivery (e.g., dual practice in public and private sectors) • Inflexible care models and “one-size-fits-all” approaches that fail to meet diverse patient needs • Primary care physicians functioning more as gatekeepers than problem solvers • Inconsistent care pathway implementation due to geographic disparities in resources • Limited investment in diagnostics, reducing access and quality	• Public-private partnerships with aligned incentives and shared care responsibilities • Interoperability between systems to support smooth patient transitions and reduce fragmentation • Upgraded facilities and care environments to improve public trust, accessibility, and efficiency
Digital Infrastructure, Technology and Data	• Clinicians face technology and data overload without adequate time, support, or training • Lack of interoperability of EHRs across care levels. • Digital exclusion among older and underserved populations lacking access to devices or skills • Limited health and digital literacy, leaving patients struggling to use tools or understand health information	• Telehealth, AI tools, and remote monitoring solutions within interoperable digital systems • Robust digital governance, including data-sharing protocols, privacy protections, and patient ownership frameworks • Regionally implemented technologies such as diagnostic hubs and registries to enhance preventive care and early detection • Effective IT systems and technical support for staff, alongside sufficient training
Communication, Engagement, and Change Management	• Limited understanding of patient preferences, with some unwilling to take ownership of their care. • Patient perceptions of GPs and community care as inferior to hospital consultants. • Stigma and discrimination surrounding NCDs such as obesity, liver disease, and mental illness. • Lack of trust in public services due to inconsistent experiences, data concerns, or past failures. • Difficulty scaling innovations, with pilots rarely moving to national implementation. • Economic analyses seldom used to guide systemic reforms or long-term planning. • Minimal monitoring of patient outcomes, experiences, or system-wide impacts.	• Political, financial, and clinical stakeholders aligned around shared goals and performance metrics • Mechanisms for meaningful engagement of communities, civil society, NGOs, community-based organisations, and private sector entities in service design and delivery to build trust and integrated responses • Shared decision-making, personalised care planning, and behavioural nudges to support self-management • Transparent communication around the evidence behind new technologies and treatments • Support for culturally relevant health literacy and engagement campaigns • Sufficient clinician training to ensure buy-in for their role in prevention. • GPs and primary hubs empowered with long-term funding and service redesign • Tailored interventions to accommodate varying literacy and comfort levels with digital tools, avoiding patient overwhelm

### 4.1 Key drivers of reform stagnation and mobilization

By identifying a distinct architecture of barriers and enablers, our findings clarify not only why change often stalls but also what is required for system-level traction. A recurrent “bottleneck” arises from funding shortages (62), insufficient and unstable political commitment (63), and deficits in digital skills and infrastructure (64). Without foundational investment in capital and human capacity, decentralisation, and health system transformation more broadly, remains largely aspirational. These hurdles are not insurmountable: robust governance and incentive models help align priorities, while proactive stakeholder management and visionary leadership are pivotal for navigating the cultural and operational complexities of reform. Sustainable change therefore depends on synchronising durable, high-level policy with practical, ground-level tools, including clear pathways, interoperable data, and workforce capability.

### 4.2 Matching reforms to local realities

The comparative case analysis underscores the importance of context. The Attend Anywhere/Near Me programme in Scotland advanced decentralised care by pairing strong national policy with programme support, local resources, and timely monitoring, conditions that accelerated spread and organisational adoption (38). In contrast, England's virtual outpatient clinic example in the Topol review encountered data gaps, capability constraints, and limited alignment with scheduling and governance, alongside resistance to co-design and technological scepticism, which likely slowed behaviour change (65). Similarly, the Danish randomised controlled trial in COPD illustrates that even well-designed remote models may not achieve intended outcomes when disease trajectories are less predictable or when adoption burdens are high (66). These contrasts highlight that strategies must be adaptive to local readiness, infrastructure, and clinical fit.

### 4.3 Institutional and structural success factors

Across cases, three intervention families were most consistently associated with positive outcomes. First, remote monitoring/virtual care models tended to reduce hospitalisations, improve quality of life, and achieve cost savings or cost neutrality when they were built on clear clinical parameters, structured high-frequency data, well-defined escalation protocols, and strong patient education components (33, 36–38, 67). Second, community-based diagnostics and screening were effective where simple diagnostic criteria, strong primary-care integration, single-point access, and geographically distributed sites streamlined pathways (27, 53, 58, 59, 67). Finally, integrated or multidisciplinary community hubs performed well when clear governance, co-location (or tight virtual coordination), and comprehensive team composition supported continuity and accountability (39, 48, 68, 69) pointing to the critical role of governance design and organisational alignment in enabling scalable and sustainable transformation.

### 4.4 NCD characteristics enabling reform

The characteristics of specific conditions further shapes the propensity for success. For example, diabetes, heart failure, cancer follow-up, and ophthalmology align well with decentralised models because they lend themselves to predictable follow-up, measurable biomarkers, image-based triage, and protocolised escalation (32, 40, 70, 71). In diabetes, behavioural elements respond to digital tools; in heart failure, daily vitals and structured response protocols enable safe remote oversight; in cancer follow-up, remote symptom monitoring reduces routine visits; and in ophthalmology, high-volume, image-based triage supports digital sorting. By contrast, mental health (given reliance on workforce availability and crisis pathways) and rare or unpredictable conditions (where clinicians perceive remote models as less safe) show more variable results. These empirical patterns converge on five universal characteristics of successful transformation: (i) predictability in clinical trajectories; (ii) standardisation of processes and pathways; (iii) substitutability (new models replace, rather than add to, existing activity); (iv) proximity and access through community locations and digital front doors; and (v) integration rather than fragmentation, with interoperable data, clear escalation routes, and shared accountability which together define the foundational conditions for successful transformation.

### 4.5 Interdependent focal areas

The three focal domains of reform, service-delivery decentralisation, workforce adaptation, and screening/early treatment, are tightly interdependent. Shifts in one domain reverberate through the others, so piecemeal change risks underperformance. The cases identified here also suggest differing time-to-impact profiles: screening initiatives often demonstrate quicker measurable outcomes; workforce policy and pipeline reforms typically manifest benefits over longer horizons; and decentralisation sits between, with some models reporting earlier gains, particularly when they embed a screening or targeted case-finding component (e.g., the Targeted Lung Health Check Programme (72),. Similarly, service delivery decentralization studies are more likely to report barriers and enablers. This could be because the projects are more complex, context-specific reforms that can expose both friction in systems as well as success factors.

### 4.6 Updating health system reform frameworks

While foundational frameworks, such as the WHO Operational Framework for Primary Health Care (2020) (73) and retrospective syntheses by Maniatopoulos et al. (2020) (74), have established the core levers for health system reform, there remains a notable gap in understanding their operationalization in the post-pandemic landscape. This study advances the evidence base by augmenting these high-level frameworks with primary stakeholder data, moving beyond theoretical abstractions to provide empirical validation. For instance, while the WHO's (2020) vision identifies leadership as a primary enabler, our findings extend this by emphasizing the necessity of policy continuity through bipartisan health strategies that transcend political cycles, a macro-political dimension often under-explored in the organizational-centric approach of Teo et al. (75). By bridging the divide between design theory and the granular realities of frontline implementation, this study offers a necessary evolution of the existing evidence base thereby supporting the identification of actionable recommendations for health system transformation. On a local level, the recommendations identified here are closely aligned with prominent national strategies, such as the NHS Fit for the Future Plan (76), which similarly underscores the necessity of transitioning from analogue to digital systems as a catalyst for change. A central tenet of our findings is the imperative to invest in robust digital infrastructure and establish interoperable systems, echoing the priorities outlined in the NHS plan, so that health systems are better equipped to implement and sustain transformative improvements.

### 4.7 Practical application

Globally, these findings provide an empirical support for the WHO Global Action Plan for the Prevention and Control of NCDs 2023–2030 (77). While the WHO framework identifies a set of cost-effective interventions for prevention, early detection, and management, it also emphasises the need to strengthen primary care systems, expand workforce capacity through task-sharing, and improve access to screening and early diagnosis at the community level. In this context, our study complements the WHO approach by identifying the system-level enablers required to operationalise these priorities in practice. By demonstrating that workforce shortages and capacity constraints act as cross-cutting inhibitors to both national transformation efforts and global NCD targets, this study underscores the need for coordinated, cross-sectoral strategies that integrate digital infrastructure, workforce adaptation, and decentralised service delivery.

This alignment with national and global strategies is not always reflected in how transformation unfolds in practice. Notably, many initiatives (outside screening and early treatment) emerged through bottom-up innovation, often in response to local triggers or disruptions rather than as part of a planned, system-wide effort. For example, in Switzerland, disruptions to local transport infrastructure prompted the development of Hospital at Home services (78). While such locally driven innovations can deliver context-specific benefits, they may also introduce variability and expose system fragilities when not supported by broader policy frameworks. Ensuring that the necessary enablers are in place at a system level may reduce reliance on reactive implementation and support the scaling and sustainability of effective models of care.

Taken together, the evidence indicates that progress hinges on pairing durable governance and financing with operational readiness: integrated pathways, interoperable digital systems, evaluation and feedback loops, and a capable, multidisciplinary workforce. Where these elements align, and where condition profiles permit predictability, decentralised and digitally enabled models can deliver meaningful gains in efficiency, equity, and patient choice.

### 4.8 Policy recommendations

**Figure 5:**
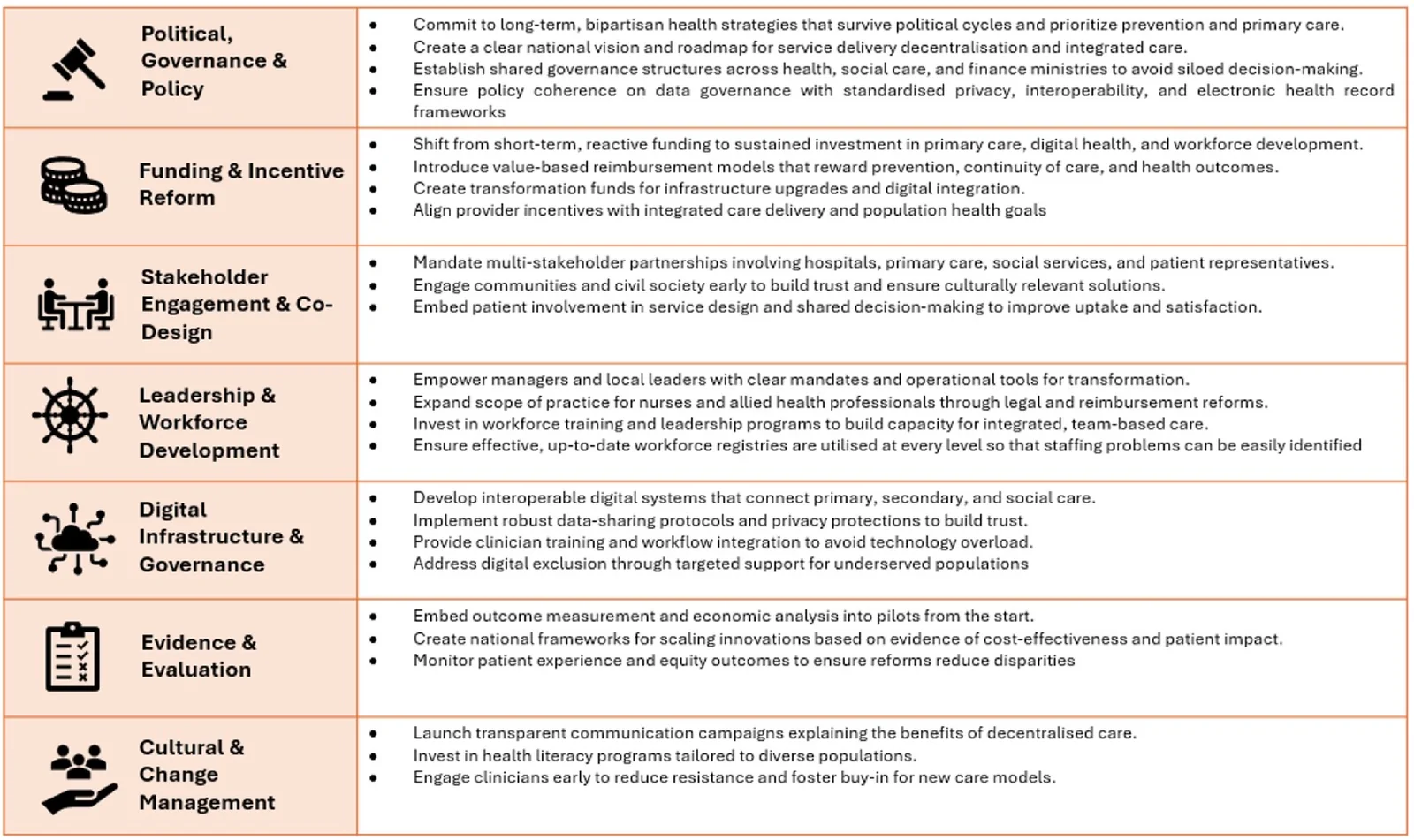
Infographic with seven categories for healthcare system transformation, each paired with an icon and action points: Political, Governance & Policy; Funding & Incentive Reform; Stakeholder Engagement & Co-Design; Leadership & Workforce Development; Digital Infrastructure & Governance; Evidence & Evaluation; and Cultural & Change Management, each specifying steps for implementation and improvement.

With this in mind and using the enablers and barriers outlined in the current analysis, we have developed a selection of recommendations, organized into six categories including: Political, governance and policy; funding, incentives & resource allocation; workforce capacity, capability & culture; system integration, structure and care coordination; digital infrastructure, technology and data and communication, engagement and change management (Figure 5). By prioritising long-term, bipartisan strategies, investing in primary and digital care, fostering collaborative partnerships, empowering leaders and staff, implementing secure and interoperable digital systems, embedding rigorous evaluation, and promoting transparent communication and health literacy, health systems can overcome barriers and deliver integrated, patient-centred care that truly improves outcomes. However, it is important to acknowledge that implementing these recommendations is not without complexity. Addressing one barrier may trigger another, related barrier. For instance, providing digital infrastructure to enable telehealth is a necessary but insufficient condition for success, its effectiveness depends equally on whether GPs are adequately trained to use these tools and on the extent to which clinicians are willing to adapt their practice. This underscores the need for iterative evaluation and adaptive management, ensuring that recommendations are not treated as one-time interventions but as evolving components of a continuously improving system.

### 4.9 Strengths and limitations

This study provides a structured synthesis of healthcare transformation processes across selected countries, focusing on case studies and reform experiences relevant to the three areas of interest. By concentrating on NCD-related disease areas, the analysis captures transformation efforts in fields associated with substantial and increasing pressures on health systems, including rising demand for services, growing complexity of care delivery, and the need for more sustainable models of prevention, diagnosis, treatment and follow-up.

A further strength of the study is its inclusion of both peer-reviewed and grey literature. This broader evidence base enabled the analysis to capture a wider range of real-world transformation initiatives than would have been identifiable through academic literature alone, particularly in cases where reforms were documented in policy reports, institutional publications, implementation materials or stakeholder-facing documents. Finally, by identifying recurring enablers and barriers across different reform areas and country contexts, the analysis supports the development of policy recommendations that are grounded in observed implementation experience and relevant to practical decision-making.

Despite these apparent strengths, this study faced several limitations that could affect the comprehensiveness of the findings. First, there was limited reporting in the literature on the actual impact of many of the healthcare reform examples analysed. Because many transformation processes take substantial time to generate observable impacts, it is not unusual for corresponding results to remain unpublished for several years. As a result, our ability to assess the effectiveness of these processes is restricted. Similarly, it is feasible to expect that there are limited publications on ‘failed’ reforms or transformation processes and that these transformation processes may have failed as a result of the presence of certain barriers. It is therefore possible that important barriers or ‘lessons learned’ may have been overlooked in the present analysis due to limited representation in the literature.

As outlined previously, this analysis focused primarily on transformation efforts and resulting barriers and enablers in OECD countries due to the impact of NCD and multimorbidity in these countries and the fact that these advanced health systems are likely to be at the forefront of solutions development. However, this focus on advanced health systems may introduce inherent bias and narrow the scope in excess, potentially excluding valuable examples of reform, and innovative enabling methodologies, from low- and middle-income countries (LMICs), many of which are dealing with rising NCD alongside a pervasive communicable disease burden and have successfully implemented transformation processes.

Finally, this study did not include a formal risk of bias assessment. We recognise the importance of assessing the methodological quality, reporting transparency and potential sources of bias in included evidence, particularly in evidence syntheses intended to inform policy recommendations. However, the evidence base included in this review was highly heterogeneous, comprising peer-reviewed studies, policy reports, institutional documents, implementation materials and other forms of grey literature. These sources varied substantially in study design, methodological reporting, analytical purpose and level of empirical detail. As a result, applying standard risk of bias tools, such as those developed for clinical trials or comparative intervention studies, would not have been appropriate or consistently feasible across the full set of included materials.

## 5 Conclusion

This analysis demonstrates that successful health-system transformation depends on the alignment of governance, financing, digital capability, and local operational readiness. It is inevitable that NCDs and other chronic conditions will place additional pressure on already overstretched and under-resourced health systems worldwide, this is largely beyond our control. However, how we choose to respond to mitigate the challenges arising from this growing burden remains firmly within our grasp. This research clearly outlines the factors that contribute to successful and sustainable reform, alongside a greater understanding of potential barriers. By combining these insights, identifying both obstacles to progress and the enablers of effective change, future reforms and policies can be designed to deliver meaningful and lasting improvements to health outcomes.

## Data Availability

The original contributions presented in the study are included in the article/Supplementary Material, further inquiries can be directed to the corresponding author.

## Footnotes

^1^Multimorbidity is defined as the co-existence of two or more chronic conditions in an individual.
