# Supplementary material for: Barriers and enablers for health system reform
Source: Front Health Serv. 2026 Sep 7;6:1887541. doi: 10.3389/frhs.2026.1887541 (PMC13599400; doi:10.3389/frhs.2026.1887541)
Supplement: Supplementary file 1 [file Table1.docx]

**Supplementary Material**

**Semi-structured Interview Guide:**

**CONTEXT AND CURRENT STATE**

1. **Current state of their health system**
   a. Could you briefly describe the healthcare landscape in your country/region, especially regarding NCD care?
2. **NCD Burden and System Preparedness**
   a. Do you think the health system in your country is well-equipped to address the burden of NCDs?
   b. In your experience, what are the most significant obstacles people living with NCDs encounter when trying to access effective care and medicines to effectively manage their condition in [country of expertise]?
3. **Drivers and Obstacles of HST**
   a. What do you see as the main drivers pushing for health system transformation?
   b. How do you think health systems need to transform to deal with these challenges?
   c. What are the biggest obstacles to transformation processes?
4. **High-level priorities**
   a. If you had to pick one or two high-priority areas for health system reform, what would they be and why?

FOCUS AREA A - DECENTRALISATION

1. **Current level of decentralisation**
a. When thinking about the current health system in [country of expertise] would you say there was a major reliance on hospital care or is a lot of health care provided in community or primary care settings?
2. **Benefits & Barriers**
a. What do you see as the main benefits (if any) of decentralising care for patients and providers?
b. Do you see any key barriers or risks with shifting care to primary or community care settings?
c. How can stakeholder engagement influence transformation efforts?
d. Does the healthcare system in [country of expertise] have the necessary capacity, resources, and infrastructure to effectively implement decentralisation?
e. Have you witnessed resistance to change from health care professionals? And if so, how do you think this can be addressed?
3. **Financing & Incentives**
a. Are there any financial or reimbursement models in your setting that incentivise community-based care, improved NCD care (i.e. collaborative care) or primary care?
4. **Coordination & Data sharing**
   a. How do providers at different levels of care communicate or share data? Are there interoperability challenges? Are health care records shared across all levels of care and across geographies?
   b. For HCPs: What do you need to collaborate effectively across the continuum of care?
5. **Decentralisation Case Study Examples**
   a. Have you been involved in, or are you aware of any pilot or policy initiative that has attempted to decentralise care (i.e. hospital at home, telemedicine, community care)?

IF YES – move onto specific case study questions

**Case study questions:**

1. Description of the initiative
   a. What triggered it, when, where, disease focus, stakeholders involved, how was it implemented.
2. Barriers
   a. Political, financial, cultural, regulatory obstacles, stakeholders, technology
3. Enablers
   a. What factors made the initiative possible or successful (e.g. leadership, community engagement, external funding)?
4. Outcome
   a. What were the measured endpoints?
   b. What outcomes were observed?
   c. What lessons were learned?
5. Scalability
   a. Do you think this could be replicated at a larger scale, or in different contexts?

FOCUS AREA B – WORKFORCE TRANSFORMATION

1. **Workforce Challenges & Drivers**
   a. What are the most pressing workforce challenges in your setting?
2. **Workforce Planning**
a. To your knowledge, is workforce planning a key part of [their setting] approach?
b. How should workforce planning be integrated into broader health system transformation strategies?
3. **Training, Upskilling & Retention**
a. What are the most critical skills healthcare workers need to develop for future health system demands?
b. What strategies could/should be used to upskill or reskill the existing workforce?
4. **Task Shifting & Skill Mix**
a. How do you view the potential for task shifting or expanding scopes of practise (e.g. pharmacists being able to prescribe antibiotics) to improve access and reduce the burden on health care practitioners?
5. **Policy Changes and Enablers**
a. In your opinion, what policy changes are needed to ensure a sustainable and resilient healthcare workforce?
b. How can regulatory and policy frameworks support the safe and effective expansion of healthcare roles?
c. Do you believe there are any other essential enablers for effective workforce provision?
6. **Key Barriers**
a. What are the biggest barriers to workforce reform (e.g., political, financial, cultural, regulatory)
b. How can stakeholder engagement (e.g., patients, providers, payers, policymakers) influence transformation efforts, if at all?
c. How do you address resistance to change? For example among healthcare professionals and institutions as well as patients?
7. **Workforce Case Study Examples**
a. Have you been involved in, or are you aware of any pilot or policy initiative that has covered any of the workforce issues we have talked about?

IF YES – move onto specific case study questions

FOCUS AREA C – SCREENING, EARLY DIAGNOSIS AND TREATMENT

1. **Current Screening Programs**
   a. Does your health system currently have National Disease Plans in place for major diseases (e.g., cancer, cardiovascular diseases, diabetes)?
   b. What are the primary screening programs implemented in your setting?
   c. What are the key components of a national screening plan, and how do they contribute to early disease detection?
   d. How are these programs structured, and what methodologies do they use?
2. **Effectiveness and Impact**
a. How effective have these screening programs been in detecting diseases at an early stage?
b. What measurable outcomes have been observed (e.g., early diagnosis rates, reduction in late-stage diagnoses, treatment success rates)?
3. **Advances in Personalized Screening and Treatment**
   a. How are advances in personalised medicine, such as genetic screening and risk stratification, being integrated into public health screening programs?
4. **Global Monitoring Frameworks**
   a. How do global monitoring frameworks for diseases like diabetes enhance international cooperation and standardization in disease management and prevention strategies?
5. **Community and Patient Engagement**
a. How are patients and communities engaged in screening programs? What strategies have been used to improve participation and awareness?
b. How do health literacy campaigns improve participation in screening programs?
6. **Integration with Primary Care**
   a. How well are screening programs integrated into primary care and community health settings?
   b. Are there effective referral pathways from diagnosis as a result of screening and treatment?
7. **Patient Navigation Programs**
   a. Are there patient navigation programs in your country? If so, is there evidence of them reducing barriers to timely cancer diagnosis and treatment, as well as improving health equity by enabling underserved populations to navigate the health system?
8. **Use of Technology in Screening**
a. Are digital tools (e.g., AI, telemedicine, decision support systems) being used to enhance screening accuracy and efficiency? What impact have they had on program effectiveness?
b. What are the primary clinical decision aid tools implemented in the country/setting?
9. **Risk Stratification Approaches**
a. How do screening programs incorporate risk stratification to target high-risk populations? (i.e. cervical screening programs, breast cancer screening programs)
b. Are there innovations being used to improve risk prediction and early detection?
10. **Enablers & Barriers**
    a. What are the key barriers to expanding screening and early treatment programs? How do factors like funding, technology availability, workforce capacity, and resistance from healthcare professionals influence their implementation?
    b. How can stakeholder engagement support the expansion and effectiveness of these programs? Do primary and community-based care providers have the necessary capacity, resources, and training to implement them successfully?
    c. What strategies have been effective in overcoming resistance to new screening technologies or early treatment models, if any?
11. **Screening Case Study Examples**
a. Have you been involved in, or are you aware of any pilot or policy initiative that has covered any of the screening/early treatment issues we have talked about?

IF YES – move onto specific case study questions

**CROSS-CUTTING ROLE OF DIGITAL TECHNOLOGY IN THE HEALTH SYSTEM**

1. Do you believe digital technologies play a role in health system transformation? If so, what kind of impact do you think they will have?
2. Do you view any digital aspects as essential pre-requisites for transformation?

**FUTURE DIRECTIONS & POLICY RECOMMENDATIONS**

1. **Key reforms**
   a. If you could implement one or two major reforms to improve NCD management and overall health system resilience, what would it be and why?
2. **Sustainability and long-term vision**
   a. What do you see as essential for sustainable HST (funding, regulation, stakeholder involvement, cross-sector collaboration)?
3. **Global trends**
   a. Are there any global trends (AI, telehealth, genetic technologies) you believe will shape the future of health systems, good or bad?
4. **Advice for key stakeholders**
   a. What advice would you offer policymakers, providers, or other stakeholders looking to accelerate HST in their contexts?

**WRAPUP AND NEXT STEPS**

1. **Final reflections -** Is there anything else you would like to add about health system transformation or any other key areas we have not covered?

**Literature Review Search Terms**

The following search terms were used in the literature analysis:

**Service Delivery Decentralisation**

("health system transformation" or "healthcare transformation" or "health reform" or "system reorganization" or "healthcare delivery reform" or "health policy change" or "healthcare restructuring" or "health system reform" or "service delivery innovation" or "integrated care reform" or "health sector reform")

AND (decentralization or "care decentralization" or "community-based care" or "localization care" or "distributed care" or "devolution healthcare" or "regional care" or "out-of-hospital care" or "primary care enhancement" or "integrated community care" or "rural health expansion" or "patient-centered care" or "community health initiatives" or "home-based care" or "remote care services" or "local healthcare access" or "healthcare delivery optimi#a*")

AND ("chronic disease management" or "non-communicable diseases" or "chronic conditions" or oncology or "cardiovascular health" or "pulmonary health" or "Ophthalmology" or "Immunology" or "diabetes" or "renal" or "Alzheimers" or "multiple sclerosis" or "Obesity")

AND ("United States" or US or "United Kingdom" or UK or Japan or Germany or France or Italy or Spain or Canada or Mexico or Korea or Colombia or OECD or "high-income countries" or "developed countries")

**Screening, Early Diagnosis and Treatment**

("health system transformation" or "healthcare transformation" or "health reform" or "system reorganization" or "healthcare delivery reform" or "health policy change" or "healthcare restructuring" or "health system reform" or "service delivery innovation" or "integrated care reform" or "health sector reform")

AND ("screening" or "early detection" or "early diagnosis" or "timely diagnosis" or "early intervention" or "treatment initiation" or "early treatment" or "early management" or "preventative screening" or "early-stage detection" or "early disease identification" or "initial diagnosis" or "preventative intervention" or "early therapeutic intervention" or "early access to care" or "pre-emptive diagnosis")

AND ("chronic disease management" or "non-communicable diseases" or "chronic conditions" or oncology or "cardiovascular health" or "pulmonary health" or "Ophthalmology" or "Immunology" or "diabetes" or "renal" or "Alzheimers" or "multiple sclerosis" or "Obesity")

AND ("United States" or US or "United Kingdom" or UK or Japan or Germany or France or Italy or Spain or Canada or Mexico or Korea or Colombia or OECD or "high-income countries" or "developed countries")

**Workforce Adaptation**

("health system transformation" or "healthcare transformation" or "health reform" or "system reorganization" or "healthcare delivery reform" or "health policy change" or "healthcare restructuring" or "health system reform" or "service delivery innovation" or "integrated care reform" or "health sector reform")

AND ("health workforce" or "healthcare workforce" or "health professionals" or "healthcare staffing" or "health workforce planning" or "health workforce projection" or " workforce transformation" or "health workforce capacity" or "health workforce shortages" or "health workforce distribution" or "workforce task shifting" or "upskilling" or "health worker retention" or "health workforce training" or "health workforce development" or "health workforce resilience" or "health worker capacity building" or " worker distribution") AND (upskilling" or "capacity building" or "retention" or "distribution improvement" or "task-shifting)

AND ("chronic disease management" or "non-communicable diseases" or "chronic conditions" or oncology or "cardiovascular health" or "pulmonary health" or "Ophthalmology" or "Immunology" or "diabetes" or "renal" or "Alzheimers" or "multiple sclerosis" or "Obesity")

AND ("United States" or US or "United Kingdom" or UK or Japan or Germany or France or Italy or Spain or Canada or Mexico or Korea or Colombia or OECD or "high-income countries" or "developed countries").

**Table 1. Interviewee details**

| Name | Country of Expertise | Affiliation |
| --- | --- | --- |
| Dr Ricardo Baptista Leite | Portugal / Europe | Policy Expert |
| Dr Eduardo Gonzalez-Pier | USA | Senior Technical Director, Palladium |
| Dr Margaret Kruk | USA | Distinguished Professor of Health Systema and Medicine, Washington University School of Medicine |
| Nicola Bedlington | UK | CEO, Millwater Partners |
| Dr Suzanne Wait | UK / Global | Co-Founder, The Health Policy Partnership |
| Anonymous | Switzerland / Europe | Health Policy Expert |
| Dr Pamela Cipriano | USA | President, International Council of Nurses |
| Anonymous | UK | Health Policy Expert |
| Mr Benedict Stanberry | UK | Director, Institute for Healthcare Leadership and Management |
| Dr Vikrant Kanwar | India | Assistant Professor of Hospital Administration, AIIMS |
| Anonymous | UK | Health Services Expert |
| Anonymous | Global | Clinician and Health Policy Expert |
| Tonya Winders | USA / Global | CEO, Global Allergy & Airways Patient Platform |
| Anonymous | Global | Clinician and Health Policy Expert |
| Gemma Allen | UK | Clinical Quality Lead, Central London Community Healthcare NHS Trust |
| Mugundhan Thottiam Parthasarathy | UK | Head of Clinical Services, Central London Community Healthcare NHS Trust |
| Anonymous | UK | Health Policy Expert |

**Case Study Policy Analysis - Harvey Balls**

Over and above the core analysis focused on identifying barriers and enablers related to case studies, subsequent analysis focused on assessing the extent to which the literature emphasised core health system objectives described in the framework – micro-efficiency, macro-efficiency, equity and choice. Each of these four core health system objectives was identified as relevant via iterative development based on themes identified across the literature and supporting evidence mapping exercises. These four areas were broken down into four subcategories, again developed following analysis of available literature and supporting evidence: (i) Micro-efficiency - care coordination; personalisation; quality of care; patient experience; (ii) Macro-efficiency - population coverage; health outcomes; system resilience; inter-sectoral benefits; (iii) Equity - geographic equity; socioeconomic equity; cultural responsiveness and inclusion of vulnerable populations; and (iv) Choice - care preferences; provider choice; treatment decisions; service options.

A set of keywords used to score case studies were then developed iteratively by translating each of the 16 sub-domains into a set of observable indicators and associated terminology. These included both direct descriptors (e.g. “integrated care”) and proxy indicators (e.g. “multidisciplinary teams”, “shared records”), informed by themes identified in the literature and supporting evidence mapping. The keywords (Table 2 in the Supplementary Material) were refined to ensure consistent coverage across domains while capturing variation in terminology across case studies. Keywords were tested in a subset of transformation examples which allowed internal review. Based on this process, keywords were added, removed, or refined to improve specificity and sensitivity. Two researchers contributed to this process with discrepancies resolved through discussion.

The refined keyword list was then used to screen evidence extracted from the case-study related literature. Specifically, a structured content analysis framework was applied, with the 16 predefined sub-categories used to code evidence extracted from the literature related to the case studies. For each sub‑category, case studies received **1 point** when there was clear supporting evidence matching typical features for that sub‑category. Total points per area were then converted to Harvey Balls scores (i.e. 0 = empty Harvey Balls, 1 = quarter, 2 = half, 3 = three-quarters and 4 = full Harvey Balls). Harvey Balls was chosen as a visual scoring tool as it provides an ‘at-a-glance’ way to show the degree of performance across the four categories.

Scoring was based on the presence of the specific keywords and phrases within the literature for each case study. For example, mention of ‘*saved bed days*’ contributed to a score for system resilience; ‘*multidisciplinary team*’ for care coordination; ‘*care closer to home*’ for geographic equity; ‘*environmental benefits*’ for inter-sectoral benefits; ‘*shared decision making*’ for treatment decisions; and ‘*patient satisfaction*’ for patient experience, as such, the resultant scores are more a measure of what is more commonly reported in the evidence, rather than a measure of the effectiveness of the transformation process. There is overlap between sub-categories, so information from studies could contribute points to multiple sub-categories across different policy areas. When studies reported mixed or absent clinical outcomes, a score was given for ‘Health Outcomes’ under Macro Efficiency only if there was clear evidence of improved outcomes (either clinical or patient-reported). If the evidence related to feasibility, usability, or process benefits, the score was instead attributed to system resilience or patient experience.

The case-study data extraction file was uploaded to Microsoft Co-Pilot (M365 Co-Pilot, based on GPT-5 chat model) and the following prompt used:

“*Score a number of case studies across 4 areas - Macro Efficiency (Population Health value) Equity (Fair Access and Outcomes); Choice (Patient Autonomy and Options) and Micro-Efficiency in a Harvey Balls manner. Each of the areas has 4 sub-categories and the case studies should be assigned 1 point (i.e 1 quarter) for each of these sub-categories they meet. The categories are as follows: 1. Macro Efficiency (Population Health value) - Population Coverage, Health outcomes, System Resilience, Inter-sectoral Benefits. 2. Equity (Fair Access and Outcomes) - Geographic equity, Socioeconomic equity, Cultural responsiveness, Vulnerable populations. 3. Choice (Patient Autonomy and Options) - Service Options, Provider Choice, Treatment decisions, Care preferences. 4. Micro-Efficiency - Care coordination, personalisation, Quality of care, Patient Experience. The relevant keywords for each are as follows:*”

A manual validation check was conducted by a researcher on ten case studies—comprising the five highest- and five lowest-scoring cases—to ensure the accuracy and consistency of the scoring.

**Table 2: Specific key-words equating to supporting evidence**

| Macro Efficiency | Population Coverage | National, nationwide, across, coverage, regions, population, 100,000, million, country-wide, EU, participants, enrolled, community healthcare networks, GP practices, screening centres |
| --- | --- | --- |
|  | Health outcomes | Reduction, reduced, improved, earlier, stage shift, mortality, quality of life, QoL, controlled, outcomes, saved lives, prevented, HbA1c, diagnosed, detected |
|  | System Resilience | Waiting times, bed days, capacity, admissions, pressure, cost-effective, efficiency, saving, workforce, resilience, reduced travel miles, carbon |
|  | Inter-sectoral Benefits | Environmental, carbon, transport costs, public-private partnership, PPP, barbers, hardware store, shopping malls, airports, train stations, voluntary sector, social care, World Bank, council, ambulance service |
| Equity | Geographic equity | Rural, remote, closer to home, care at home, home treatment, satellite unit, community centre, travel, km, kilometres, miles, decentralisation, decentralization |
|  | Socioeconomic equity | Low-income, deprivation, underserved, marginalized, equity, free service, affordable, cost saving for patients |
|  | Cultural responsiveness | Culturally responsive, First Nations, language, languages, multilingual, cultural |
|  | Vulnerable populations | Frail, older, pregnant, complications, children, vulnerable, chronic, multiple chronic conditions, MCC, mental, psychiatric |
| Choice | Service Options | Face-to-face, digital, virtual, home monitoring, self-sampling, walk-in, mobile practice, video consultations, opt-in, opt-out, primary care and hospital, telemonitoring |
|  | Provider Choice | Optometry, primary eye care, GP and community, provider, network of providers, multiple providers, across practices |
|  | Treatment decisions | Shared decision, self-management, engage, self-care advice, treatment plan |
|  | Care preferences | Preference, prefer, stay home, home administration, patient experience, comfort at home |
| Micro-Efficiency | Care coordination | Multidisciplinary, coordination, co-ordinated, navigator nurses, integrated, pathways, care coordinator |
|  | Personalisation | Personalised, personalized, genomic, individual, iPDM (integrated personalised diabetes management), risk calculations |
|  | Quality of care | Quality of care, quality, accuracy, diagnostic, NICE guidance, improved outcomes, cost-effective |
|  | Patient Experience | Patient experience, satisfaction, empowered, acceptability, well-being, engagement, shorter, less stress |

Across the 67 case studies, none addressed all four subcategories within all four policy categories analysed (macro-efficiency, micro-efficiency, equity, and choice). Thus, no study achieved a perfect score for the breadth of information covered (Table 3 in Supplementary material). Only six of the case studies achieved a full score of four in any single area – all within macro efficiency and choice (Table 4 in the Supplementary material). Case studies tended to achieve a higher score for macro efficiency than for the three other areas, suggesting that transformation efforts are more focused on reporting outcomes related to macro efficiency – ‘*population coverage*’, ‘*health outcomes*’, ‘*system resilience*’ and ‘*inter-sectoral benefits*’ – and less focused on reporting issues related to equity, choice and micro efficiency. Although it is important to note that score achieved in each area is related to the specific references used and the breadth of extracted evidence and does not necessarily equate to the actual achievement of the policy goals.

**Table 3: Case study details - Harvey Balls Analysis Results**

| Name | Type^*^ | Micro-Efficiency | Macro Efficiency | Equity | Choice | Reference |
| --- | --- | --- | --- | --- | --- | --- |
| 24/7 remote rural health monitoring | D | ◑ | ◕ | ◑ | ◔ | (Ang, 2023a) |
| Attend Anywhere / Near Me video consulting service evaluation 2019-2020: report | D | ◑ | ● | ◕ | ◑ | (Wherton and Greenhalgh, 2020) |
| Benefits and Challenges of Remote Patient Monitoring as Perceived by Health Care Practitioners: A Systematic Review | D | ◑ | ◔ | ◕ | ◔ | (Serrano et al., 2023) |
| Canada’s primary care crisis: Federal government response | D | ◔ | ◔ | ◔ | ◑ | (Flood et al., 2023) |
| CLCH leads pilot study to bring COPD and asthma management to patients in their own homes | D | ◑ | ◔ | ◔ | ○ | (NHS CLCH, 2022a) |
| Clinical impact of an integrated e-health system for diabetes self-management support and shared decision making (POWER2DM) | D | ◕ | ◑ | ◑ | ◔ | (Ruissen et al., 2023) |
| Chronically ill people need well-developed primary care, as a simulation model shows | D | ◔ | ◕ | ◔ | ◑ | (Schonenberger, 2019) |
| Community Eye Centres (CEC) - Decentralisation of an Ophthalmology Service | D | ◕ | ◕ | ◔ | ◑ | (Conclude Informed Decision Making, 2014) |
| Decentralisation of radiation therapy - RUTE satellite radiotherapy unit | D | ◔ | ● | ◔ | ○ | (Arenas et al., 2015) |
| Digital Transformation in Ophthalmic Clinical Care During the COVID-19 Pandemic | D | ○ | ◕ | ◑ | ◑ | (Kim et al., 2021) |
| Effectiveness of telemonitoring to manage COPD: A systematic review | D | ◑ | ◔ | ○ | ● | (Kruse et al., 2019) |
| Enhanced Community Care | D | ◑ | ● | ◕ | ○ | (HSE, 2025) |
| Feasibility of health-related quality of life (HRQoL) assessment for cancer patients using electronic patient-reported outcome (ePRO) in daily clinical practice (QOLIBRY study) | D | ○ | ◔ | ○ | ○ | (Mouillet et al., 2021) |
| GP Pathfinder Clinics | D | ○ | ○ | ○ | ◔ | (“GP Pathfinder Clinics,” 2026) |
| Home monitoring for women with pregnancy complications – better, closer, and more personal care -sing Nemo Healthcare CTG devices | D | ○ | ◑ | ◑ | ◑ | (Eindhoven University of Technology, 2022) |
| Hospital at Home | D | ◑ | ◑ | ◑ | ◑ | (NHS CLCH, 2022b) |
| Hospital at Home service - Spital Zollikerberg Zuhause | D | ○ | ◑ | ○ | ○ | (Spital Zollikerberg, 2025) |
| House of Health of Bettola- Reducing the burden of travel and environmental impact through decentralization of cancer care | D | ○ | ◕ | ◔ | ○ | (Cavanna, 2024) |
| How to improve care coordination for people with chronic conditions in Switzerland? | D | ◑ | ◑ | ◔ | ◔ | (Swiss National Science Foundation, 2019) |
| Optometry First Toolkit | D | ◑ | ● | ○ | ◔ | (NHS, 2022) |
| Person-Centered Care Planning (PCCP) for People Living With or at Risk for Multiple Chronic Conditions | D | ◑ | ◕ | ◔ | ◑ | (Watson et al., 2024) |
| Ping An Good Doctor one minute clinics | D | ○ | ◑ | ◔ | ◔ | (MobiHealth News, 2019) |
| Remote monitoring in heart failure care | D | ○ | ◕ | ◔ | ○ | (Koehler et al., 2018) |
| [Safe@home](mailto:Safe@home) | D | ◔ | ◕ | ◑ | ◑ | (Ang, 2023b) |
| Self-management and remote monitoring in prostate cancer | D | ◔ | ◑ | ◔ | ◑ | (Frankland et al., 2019) |
| South Australian Child and Adolescent Virtual Urgent Care | D | ○ | ◑ | ◔ | ◔ | (Women’s and Children’s Health, 2026) |
| Subcutaneous (s.c.) administration of bortezomib for multiple myeloma at home | D | ◑ | ◑ | ○ | ◔ | (Lassalle et al., 2016) |
| Targeted lung health check programme (TLHC) | D | ○ | ● | ◔ | ◔ | (NHS England, 2019) |
| Telemonitoring for COPD patients - quality of life study | D | ○ | ◑ | ○ | ◔ | (Køpfli et al., 2023) |
| The PDM-ProValue study program [4] | D | ◕ | ◑ | ○ | ○ | (Kulzer et al., 2018) |
| The right care in the right place (JZOJP) | D | ◔ | ◕ | ◔ | ◔ | (Care in the right place Taskforce, 2018) |
| Treating people with acute mental illnesses at home | D | ◔ | ◔ | ◑ | ○ | (Swiss National Science Foundation, 2016) |
| Trial to monitor chemotherapy side effects remotely | D | ◔ | ◑ | ◔ | ◔ | (Maguire et al., 2021) |
| Virtual outpatient clinic development: using the NASSS framework | D | ◔ | ◔ | ◔ | ○ | (Topol, 2019) |
| Virtual wards | D | ◑ | ◕ | ◔ | ◔ | (Hutchings and Edwards, 2023) |
| We4You - Integrated medical practice | D | ◔ | ◑ | ○ | ◔ | (Medinside, 2024) |
| Digitalisation in Estonia towards disease prevention and early detection | D / S | ◑ | ◑ | ◔ | ○ | (ICPerMed, 2019) |
| Virtual clinics for people with respiratory symptoms | D / S | ◔ | ◑ | ◔ | ◔ | (Health Service Journal, 2020) |
| Blood pressure trial launches in Bunnings NSW | S | ◔ | ◕ | ○ | ○ | (George Institute of Global Health, 2024) |
| C the Signs | S | ◑ | ◔ | ○ | ○ | (NHS England, 2018) |
| Cost-Effectiveness Analysis of Colorectal Cancer Screening Strategies in Singapore: A Dynamic Decision Analytic Approach | S | ◔ | ◑ | ○ | ○ | (Wong et al., 2004) |
| Deployment of a multi-tissue AI-based quality control system in routine clinical workflow (GALEN) | S | ◔ | ◔ | ○ | ○ | (Sandbank et al, 2022) |
| Diabetes Prevention Programme (NHS DPP) | S | ◔ | ◔ | ○ | ◔ | (NHS England, 2022) |
| Digital Health check | S | ○ | ◕ | ◔ | ◔ | (Department of Health and Social Care, 2023) |
| Early diagnosis for Breast Cancer | S | ◔ | ◑ | ○ | ○ | (WHO, 2021) |
| Global Flu View | S | ◔ | ◔ | ○ | ◔ | (Crawley et al., 2019) |
| HPV screening in Scotland | S | ○ | ○ | ◔ | ○ | (Barry, 2024) |
| Lessons learnt from the implementation of a colorectal cancer screening programme for lynch syndrome in a tertiary public hospital | S | ◑ | ◑ | ○ | ○ | (Dueñas et al., 2023) |
| Lung cancer screening programme in Croatia | S | ○ | ◑ | ○ | ◔ | (Arnold, 2023) |
| Moldova: Radiology and Diagnostic Imaging Center | S | ◔ | ◑ | ○ | ◔ | (Fatur, 2012) |
| NHS blood pressure checks at the barbers to prevent killer conditions | S | ◔ | ◕ | ◔ | ○ | (England, 2023) |
| Opt-in and opt-out self-sampling HPV screening strategies | S | ◔ | ◔ | ○ | ◔ | (Huntington et al., 2024) |
| Screening Centre Integration: HPV self-sampling at mammography clinics | S | ○ | ◑ | ◔ | ◔ | (Bonde, 2025) |
| USPSTF Colorectal Cancer Screening Recommendation and Uptake for Individuals Aged 45 to 49 Years | S | ◑ | ◑ | ○ | ○ | (Siddique et al., 2024) |
| Development of a comprehensive prevention strategy for CKD in France | S / W | ◑ | ◔ | ○ | ○ | (“Nephrolor,” 2025) |
| Evaluation of the Effectiveness of Health Care Provider Incentive Programs in Oregon | W | ○ | ◑ | ◕ | ◔ | (Oregon Health Authority, 2023) |
| Interventions for health workforce retention | W | ◔ | ◑ | ○ | ○ | (Russell et al., 2021) |
| Americas Health Corps – Fuerza de Salud de las Americas | W | ◔ | ◑ | ◕ | ◔ | (PAHO, 2022) |
| BeWell project - skills development initiative | W | ◔ | ◑ | ◔ | ◑ | (BeWell, 2022) |
| Healh workforce New Zealand | W | ○ | ◑ | ○ | ○ | (Rees, 2019) |
| HOPE - Hospitals of EurOPE | W | ○ | ◔ | ○ | ○ | (European Hospital and Healthcare Federation, n.d.) |
| HR Action Plan | W | ○ | ◑ | ○ | ◔ | (Stefanson, 2022) |
| IFMSA exchange program | W | ○ | ◔ | ◔ | ◔ | (“Canadian Federation of Medical Students,” n.d.) |
| Nurse-led care models reduce unplanned hospital admissions | W | ◔ | ◕ | ◔ | ○ | (Simon, 2016) |
| Pharmacy First | W | ◔ | ◔ | ○ | ○ | (NHS England, 2024) |
| Skill mix in Poland | W | ◑ | ◕ | ◔ | ○ | (Kowalska-Bobko et al., 2021) |
| Workforce Incentive Program – Rural Advanced Skills Stream | W | ○ | ◑ | ◕ | ○ | (Australian Government Department of Health, 2025) |

Notes: * D refers to case studies focusing on service delivery decentralisation, S to those focusing on screening, W to those focusing on workforce adaptation and D/S to those that have elements of both service delivery decentralisation and screening. Scoring represents a measure of what the evidence reports.

Table 4: Proportion of case studies achieving each Harvey Ball score for each policy requirement.

| Number of case studies achieving score | Micro Efficiency | Macro Efficiency | Equity | Choice |
| --- | --- | --- | --- | --- |
| 0 (○) | 21 | 2 | 27 | 28 |
| 1 (◔) | 25 | 16 | 27 | 27 |
| 2 (◑) | 18 | 29 | 7 | 11 |
| 3 (◕) | 3 | 15 | 6 | 0 |
| 4 (●) | 0 | 5 | 0 | 1 |

Almost all case studies report at least some evidence aligned with one or more of the higher-level categories – less than 5% of the analysed studies reported *no* evidence in any of the four categories. Those that did tended to represent early pilots or descriptive profiles with limited evaluation. Only 15% of case studies scored well (i.e. two or more) across three or more categories, indicating that there is limited reporting of multi‑dimensional, system‑level transformation. Similarly, only two of the case studies scored more than two across all four higher-level dimensions – ‘Attend anywhere / Near me’ in Scotland (Wherton and Greenhalgh, 2020) and the UK based ‘Hospital at Home’(NHS CLCH, 2022b). Both examples focus on service delivery decentralisation and combine macro level improvements with real patient-level and choice gains. In fact, the ‘top 10’ scoring case study examples were all models focusing on service delivery decentralisation. These examples all clustered around virtual and / or community care models suggesting these types of case studies are more likely to have information published.
